# River export of macro- and microplastics to seas by sources worldwide

**DOI:** 10.1038/s41467-023-40501-9

**Published:** 2023-08-10

**Authors:** Maryna Strokal, Paul Vriend, Mirjam P. Bak, Carolien Kroeze, Jikke van Wijnen, Tim van Emmerik

**Affiliations:** 1 Water Systems and Global Change Group, Wageningen University, Wageningen, The Netherlands; 2 Ministry of Infrastructure and Water Management, Directorate-General for Public Works and Water Management, Utrecht, Netherlands; 3 Environmental Systems Analysis Group, Wageningen University, Wageningen, The Netherlands; 4 Department of Environmental Sciences, Faculty of Science, Open University, Heerlen, The Netherlands; 5 Hydrology and Quantitative Water Management Group, Wageningen University, Wageningen, The Netherlands

**Keywords:** Geochemistry, Element cycles

## Abstract

Seas are polluted with macro- (>5 mm) and microplastics (<5 mm). However, few studies account for both types when modeling water quality, thus limiting our understanding of the origin (e.g., basins) and sources of plastics. In this work, we model riverine macro- and microplastic exports to seas to identify their main sources in over ten thousand basins. We estimate that rivers export approximately 0.5 million tons of plastics per year worldwide. Microplastics are dominant in almost 40% of the basins in Europe, North America and Oceania, because of sewage effluents. Approximately 80% of the global population live in river basins where macroplastics are dominant because of mismanaged solid waste. These basins include many African and Asian rivers. In 10% of the basins, macro- and microplastics in seas (as mass) are equally important because of high sewage effluents and mismanaged solid waste production. Our results could be useful to prioritize reduction policies for plastics.

## Abstract (web summary)

Modelling of riverine plastic exports finds microplastics dominate in areas with many sewage systems and macroplastics where waste is mismanaged. In some areas both plastics are important. Reduction at source is needed.

## Introduction

Plastic pollution is increasing in many aquatic systems^1,2^ and is already a global concern^3^. Plastics in water can negatively impact human livelihoods and aquatic organisms and be a potential risk to human health^4–7^. Approximately 0.8–23 million tons of plastics enter seas worldwide annually^8–12^. Rivers may contain both macro- (>5 mm)^13^ and microplastics (<5 mm)^14^, but their sources are not well-studied simultaneously^7^. Most plastic pollution is produced on land and transported from land to rivers. Rivers can export plastics further to seas^13,14^. However, not all plastics reach the seas, and some plastics stay in the river system^13,15^. The number of published studies on plastic pollution has increased considerably over the past 5 years^7,16^. Nevertheless, four main knowledge gaps still exist.

The first knowledge gap is related to the interrelations between micro-and macroplastics. Previous work determined that the size of plastic particles in river export varies in time and space^17^, and macroplastics are a major source of microplastics^18^. This implies that changing macroplastics in rivers may also affect microplastic flows. These are relevant insights for policy but lacking in the current literature on global rivers. Most modelling studies, however, focus on either microplastic^4,14,19–25^ or macroplastic^8,26–28^ exported by the world’s rivers. Few models account for macro- and microplastics^29^. Often, these models are limited to rivers^9^ or specific to location and time^30,31^.

The second knowledge gap is related to the spatial origin of plastic pollution. Our understanding of where plastic pollution is produced within river basins is poor, especially within large rivers such as Mississippi (North America), Amazon (South America), Danube (Europe), Nile (Africa), and Ganges (Asia)^32^. Globally, such analyses exist for other pollution types^32^ but not for plastics. The Model to Assess River Inputs of pollutaNts to seAs (MARINA-Multi, version 1) takes a sub-basin-scale modelling approach to quantify inputs of multiple pollutants to rivers^32^. Sub-basins are defined here as sub-catchments obtained by dividing the large river basins (e.g., Mississippi, Danube and Ganges) into smaller geographical units. However, the MARINA-Multi model only accounts for microplastic from point sources (e.g., sewage systems) and does not consider macroplastics. Moreover, it only accounts for the inputs to rivers, but not for river export of plastics to coastal seas.

The third knowledge gap is related to the different sources of plastics in river sub-basins. Different human activities lead to point sources of pollution^32^ or mismanaged solid waste and diffuse littering^2,14^. Mismanaged solid waste is the largest source of macroplastics in water^2^. Important sources of microplastics in sewage are car tyre wear particles, personal care products (PCP), laundry fibres^14^, and the degradation of macroplastics^33^. Accounting for the source attribution of both macro- and microplastics is needed for prioritising effective integrated strategies to reduce sea pollution. A combined analysis of the origin (e.g., basins) and sources (e.g., sewage, waste) of macro- and microplastics in rivers and seas is scarce for both types of plastics worldwide. This challenges the formulation of strategies to reduce future plastic pollution and achieve Sustainable Development Goals (SDGs).

The fourth knowledge gap is related to the integration of information on the type of plastics, the spatial origin of the plastics, and the specific human sources of plastics. Current large-scale studies report on the contribution of individual world rivers to pollution of coastal seas with either micro- or macroplastics^9,13,34^, identifying the most polluting rivers^9,13^. However, such studies typically ignore sub-basin variability in sources of pollution and river retention. Some sub-basins might be dominantly polluted by one type of plastic pollution (macro or micro) or both plastics (macro and micro) and have different dominant sources.

In this work, we model riverine macro- and microplastic exports to seas to identify their main sources in over ten thousand sub-basins. We consider largely urban-related sources, including sewage systems for microplastics (point source) and mismanaged solid waste for macro- and microplastics (diffuse source). Sewage systems collect wastewater from streets (microplastics from car tyres) and houses (microplastics from personal care products, household dust and laundry). Mismanaged solid waste contains macroplastics that can fragment into microplastics. We develop the MARINA-Plastics model to estimate riverine exports of macro- and microplastics to seas as a function of human activities on the land (e.g., mismanaged waste, sewage and treatment) and retention rates in the rivers (e.g., along the riverbanks, plastic fragmentation, water consumption, Supplementary Fig. 1). We categorise sub-basins into three main classes based on the ratio of macro- and microplastics in riverine exports and their dominant sources (see Supplementary Note). We show that when microplastics dominate, sewage systems (point source) are often the main sources of pollution. When macroplastics dominate, mismanaged solid waste (diffuse source) is the main source of pollution. We further identify sub-basins where both macro- and microplastics are present equally, implying that both sewage systems and mismanaged solid waste are important pollution causes. These insights could be used by policymakers to prioritise the design of solutions.

## Results and discussion

### Riverine plastic export to coastal seas

**Fig. 1:**
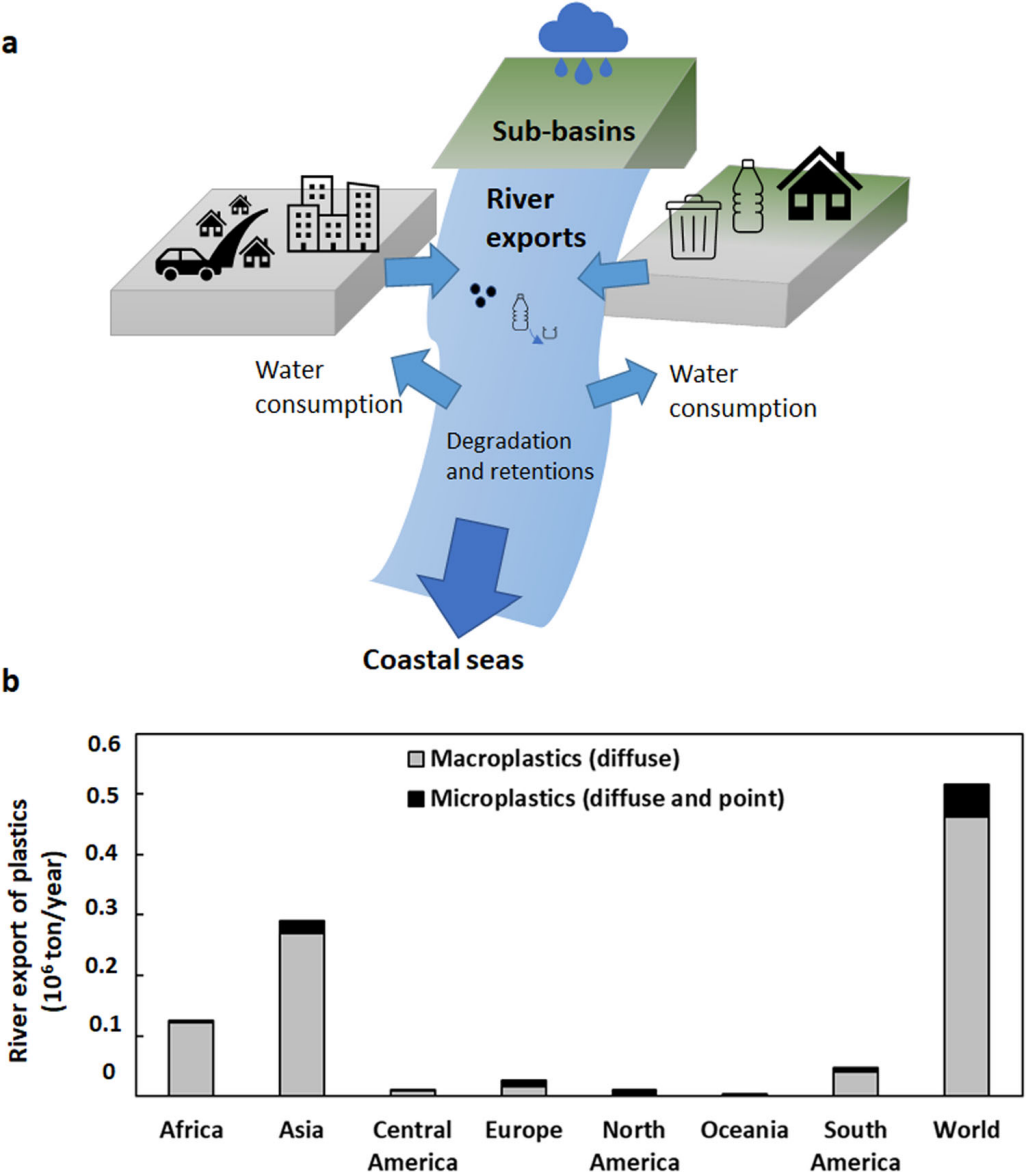
MARINA-Plastics model for riverine plastic exports to coastal seas. **a** Overview of the MARINA-Plastics model (see “Methods”). **b** The amount of macro- and microplastic export by rivers globally and by region (in mass units, 10^6^ tons/year). Sub-basins are defined as sub-catchments. Supplementary Tables 1–10 and Supplementary Figs. 1–6 provide details on the model, data and results.

**Fig. 2:**
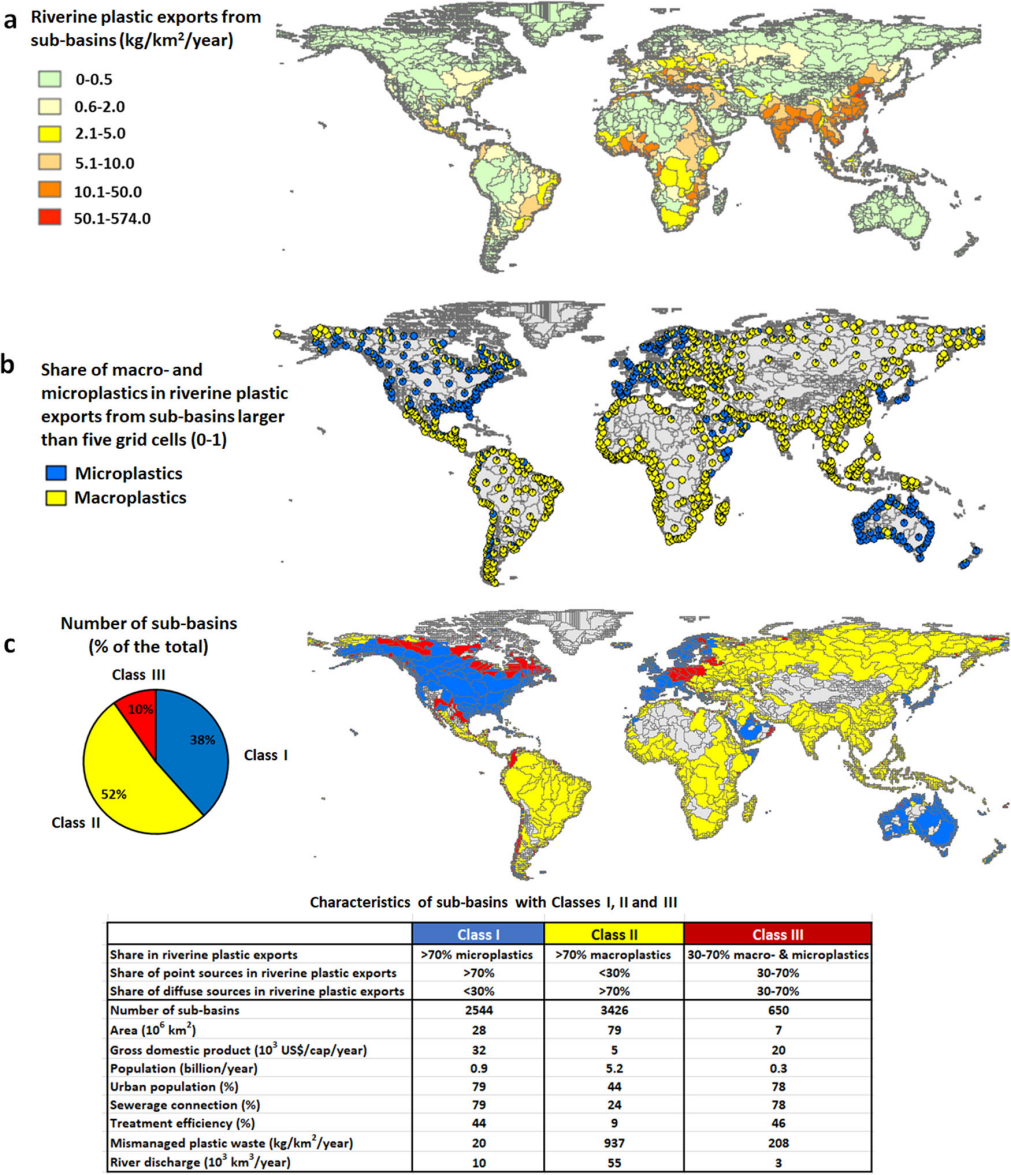
Modelled riverine plastic exports to coastal seas. **a** Total riverine plastic exports to seas (kg/km^2^ of the sub-basin area/year). **b** Relative shares of macro- and microplastics in the total river export (fraction, 0–1). **c** Classification of sub-basins in three classes: I (microplastics dominated from point sources), II (macroplastics dominated from diffuse sources), and III (mixed). The study area has 10,226 sub-basins. Of these, riverine plastics from 6620 sub-basins reach coastal seas. The other sub-basins (indicated in grey) either do not drain into coastal seas (1318 sub-basins) or export zero plastics (2288 sub-basins). In addition, Fig. 2b presents results for selected sub-basins, which drainage areas are higher than five grid cells. Pollution sources are specified in Fig. 3 and Supplementary Fig. 7. Classes are described in Supplementary Note. Source: The MARINA-Plastics model (see the “Methods” section).

Globally, rivers are estimated to export approximately 0.5 million tons of plastics to the seas per year (Fig. 1). Approximately 80% of this amount is macroplastics exported by Asian and African rivers (Fig. 1, Supplementary Fig. 2). Riverine plastic exports vary among sub-basins (Fig. 2). For example, high levels of riverine plastic export of >10 kg/km^2^/year are identified in a large fraction of the rivers in Asia, Africa, Central America, and South America. Lower riverine plastic exports are identified in sub-basins in North America and Europe (Fig. 2, Supplementary Fig. 2). We further analyse sub-basins by classifying them into three classes based on the ratio of macro- and microplastics in river exports and their sources (see Fig. 2 for the definition and Supplementary Note). Class I comprises sub-basins in which rivers export over 70% of plastics by mass as microplastics, and over 70% of this is from point sources (sewage systems, Fig. 2). Class II comprises sub-basins in which rivers export over 70% of plastics as macroplastics and over 70% of this is from diffuse sources (mismanaged solid waste). Class III includes rivers where macro- and microplastic have a more equal share (30–70%) in the total plastic export from sub-basins and both point and diffuse sources are important contributors (Fig. 2, Supplementary Note 1).

### Sub-basins where microplastic pollution dominates from point sources (Class I)

**Fig. 3:**
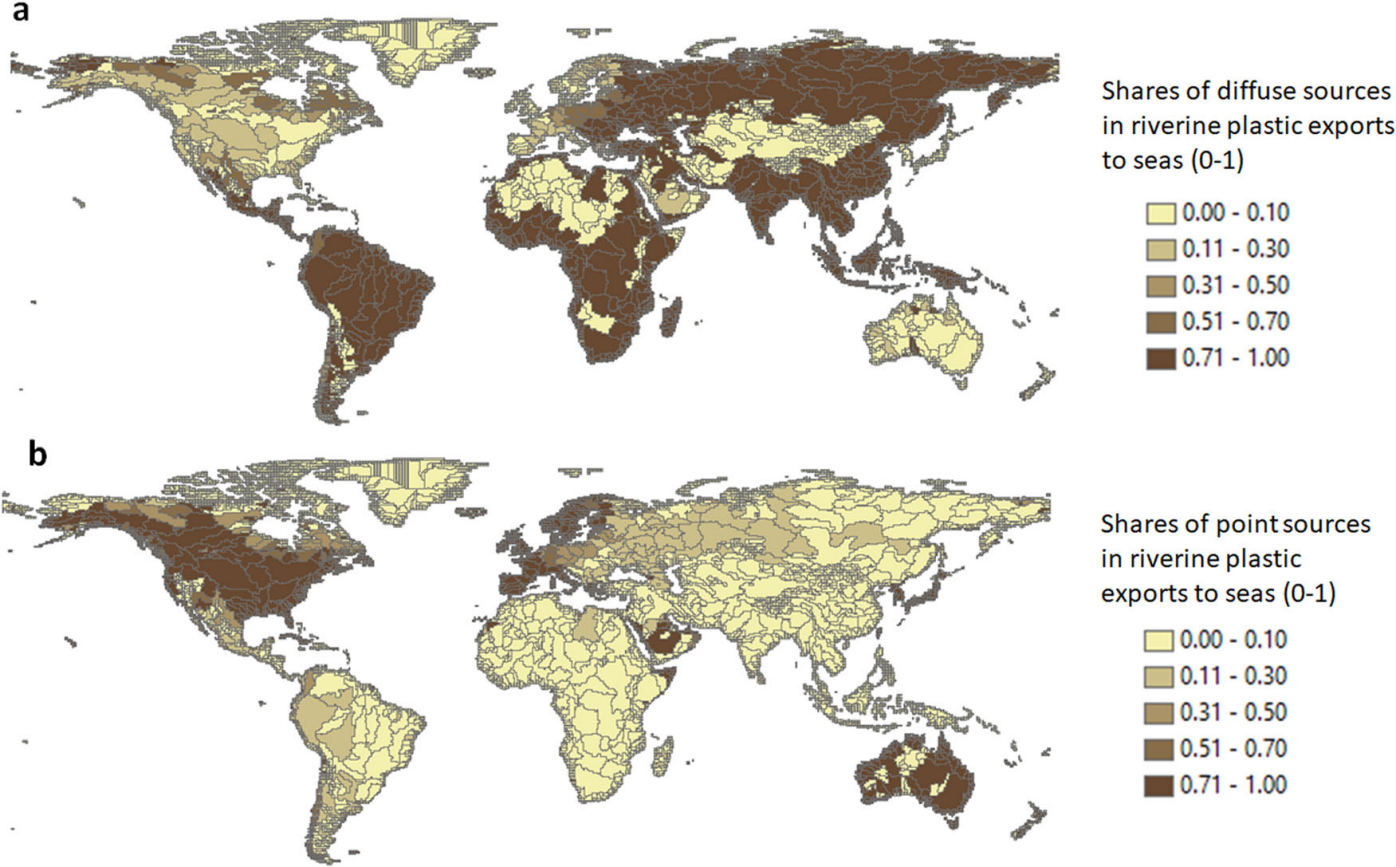
Sources of riverine plastic exports to seas from sub-basins (shares, 0–1). **a** Shares of diffuse sources including mismanaged solid waste for macroplatics and the fragmentation of macroplastics into microplastics (0–1). **b** Shares of point sources including sewage systems that discharge microplastics from car tyres, personal care products, laundry and household dust (0–1). Supplementary Fig. 7 provides more details. Source: The MARINA-Plastics model (see the “Methods” section).

**Fig. 4:**
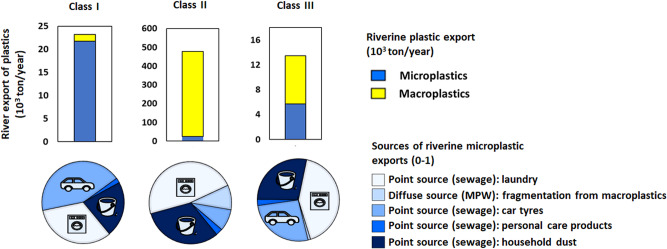
Modelled riverine plastic export from Class I–III sub-basins to coastal seas. Pie charts show model results for macro- and microplastics (10^3^ tons/year). Pies show the shares of the sources of microplastics in river export (fraction, 0–1). MPW is short for mismanaged plastic waste. Supplementary Fig. 7 provides these results for the continents. Source: The MARINA-Plastics model (see the “Methods” section).

**Fig. 5:**
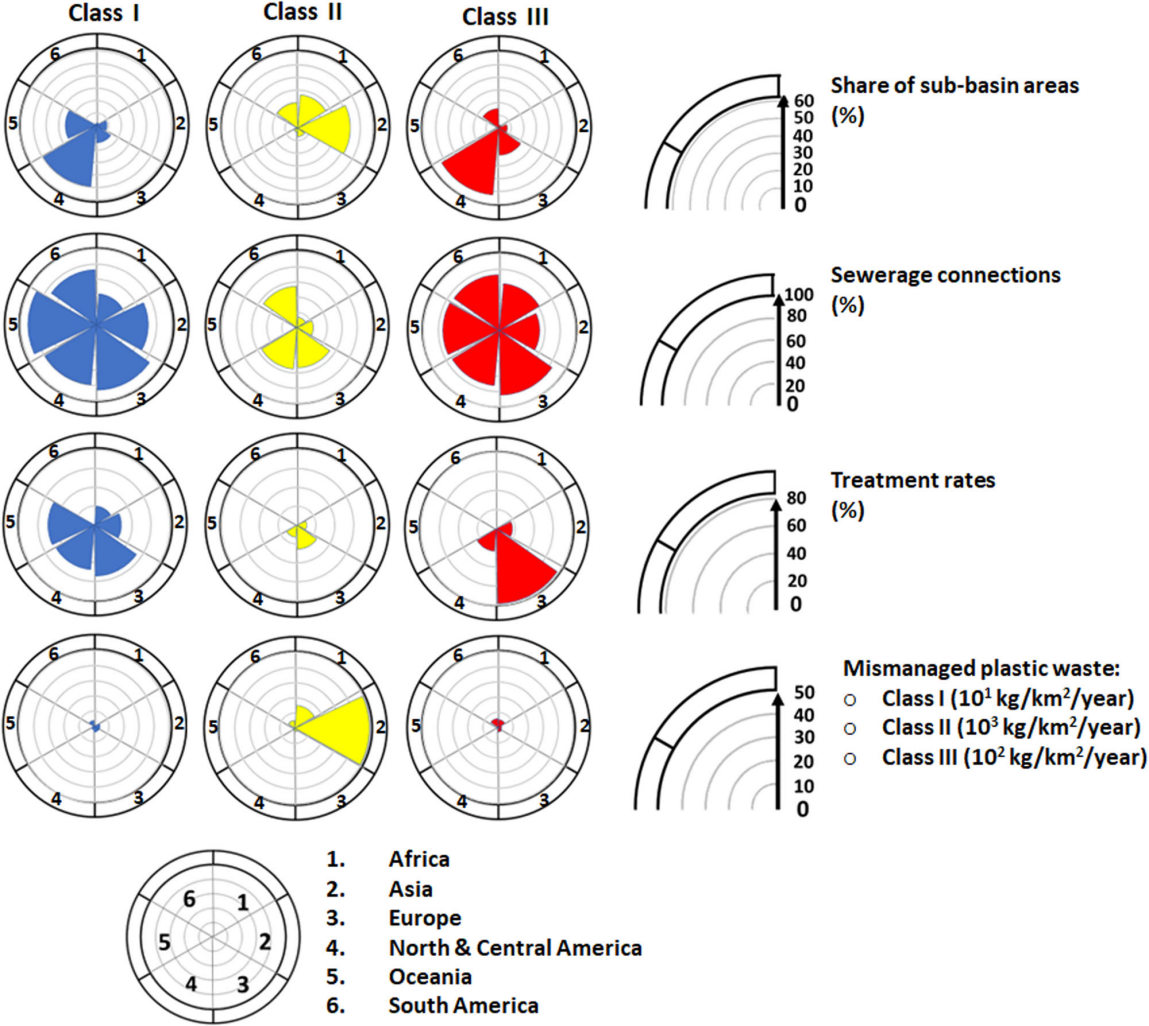
Main characteristics of Class I–III sub-basins. The classes include sub-basins from different regions (see Fig. 2 and Supplementary Fig. 2). The shares of sub-basin areas are calculated relative to the total sub-basin area in each class. Descriptions of how the sewage connection, the average treatment rates, and mismanaged plastic waste for each class are calculated are provided in the “Methods” section under “Three classes for river exports of plastics”. Source: see the “Methods” for the references to the data.

These sub-basins are largely located in Europe, North America and Oceania (Fig. 2). They make up 38% of the sub-basins globally (*n* = 2544), with a drainage area of 28 million km^2^ equalling one-fifth of the global land surface area. Rivers are modelled to export up to 25,000 tons of plastics from their sub-basins to seas annually. Over 80% of this amount is microplastic mainly from point sources, which are sewage systems with the contribution of 40% for car tyres, 31% for laundry, 21% for household dust, and 2% for personal care products (Figs. 3–4). Diffuse sources (the fragmentation from macroplastics) contribute slightly to microplastic exports (<0.1%, Fig. 4). Among sub-basins, the contribution of point sources is over 70% and for diffuse sources, this is below 30% (Fig. 3). High contributions of point sources are associated with high sewage connection rates (around 80%), moderate treatment (44% on average) and low mismanaged plastic waste in the environment (20 kg/km^2^ of sub-basin area/year, Figs. 2 and 5, Supplementary Fig. 3). However, this varies among the sub-basins that are located in different regions (Fig. 5 and Supplementary Fig. 7). Sewage connections range from 41% for the African to 91% for the Australian sub-basins (Fig. 5). Treatment rates range from 20% for the African to 54% for the European sub-basins. In the sub-basins of South America, treatment is low (<0.1%, Fig. 5).

### Sub-basins where macroplastic pollution dominates from diffuse sources (Class II)

These sub-basins are largely located in Asia, Africa, and South America (Fig. 2). They represent 52% of the sub-basins globally (*n* = 3426) with a drainage area of 79 million km^2^ equalling 70% of the global land area that accommodates approximately 80% of the global population (5.2 billion, Fig. 2). Rivers are modelled to export approximately 450 thousand tons of plastics to the seas (90% of the global riverine plastic export). Most of these are macroplastics from diffuse sources, which is mismanaged solid waste. Among sub-basins, the contribution of this diffuse source is above 70% (Figs. 3 and 4), which is largely associated with the high amount of mismanaged solid waste entering the environment (937 kg/km^2^ of sub-basin area/year, Figs. 2 and 5). These sub-basins are generally less developed (5000 US$/cap/year), with lower sewage connection rates (24% on average) and treatment (9% on average) compared to sub-basins in Class I (Figs. 2 and 5). Sewage connections range from 3% for the Oceanian to 55% for the North and Central American sub-basins (Fig. 5). This implies fewer inputs of microplastics from sewage systems (point source) in these sub-basins. In contrast, the largest source of mismanaged solid waste to the environment is in the Asia sub-basins (50,000 kg/km^2^/year), followed by the African sub-basins (15,000 kg/km^2^/year). This implies more inputs of macroplastics in these sub-basins.

### Sub-basins with similar levels of macro- and microplastic pollution from point and diffuse sources (Class III)

Class III sub-basins are located in parts of North America and Europe as well as along the coast of South America (Fig. 2). They make up 10% of the sub-basins globally (*n* = 650), with a drainage area of 28 million km^2^ equalling to 6% of the global land area (Fig. 2). Rivers are modelled to export around 13,000 tons of plastics (approximately 2% of the global riverine plastic export). Approximately 40% of this modelled amount is microplastics from point sources, and 60% is macroplastics from diffuse sources (Figs. 3 and 4). The contribution of point sources in riverine microplastic export is one-third for car tyres and two-thirds for laundry and household dust in sewage systems. All macroplastics in the sea are from mismanaged waste introduced to the environment (diffuse source, Fig. 5). These sub-basins have urbanisation and treatment rates that are similar to those in sub-basins of Class I, but accommodate fewer people (0.3 billion) compared to Classes I and II (Fig. 5). The amount of mismanaged solid waste being introduced to the environment (208 kg/km^2^/year) is compared to Class I (20 kg/km^2^/year), but lower than Class II (937 kg/km^2^/year, Fig. 2). Sewage connections range from 53% for the Asian to 85% for the European sub-basins. The range for treatment efficiencies varies from zero (Africa, Oceania, South America) to 78% (Europe, Fig. 5).

### Comparisons with other studies

**Fig. 6:**
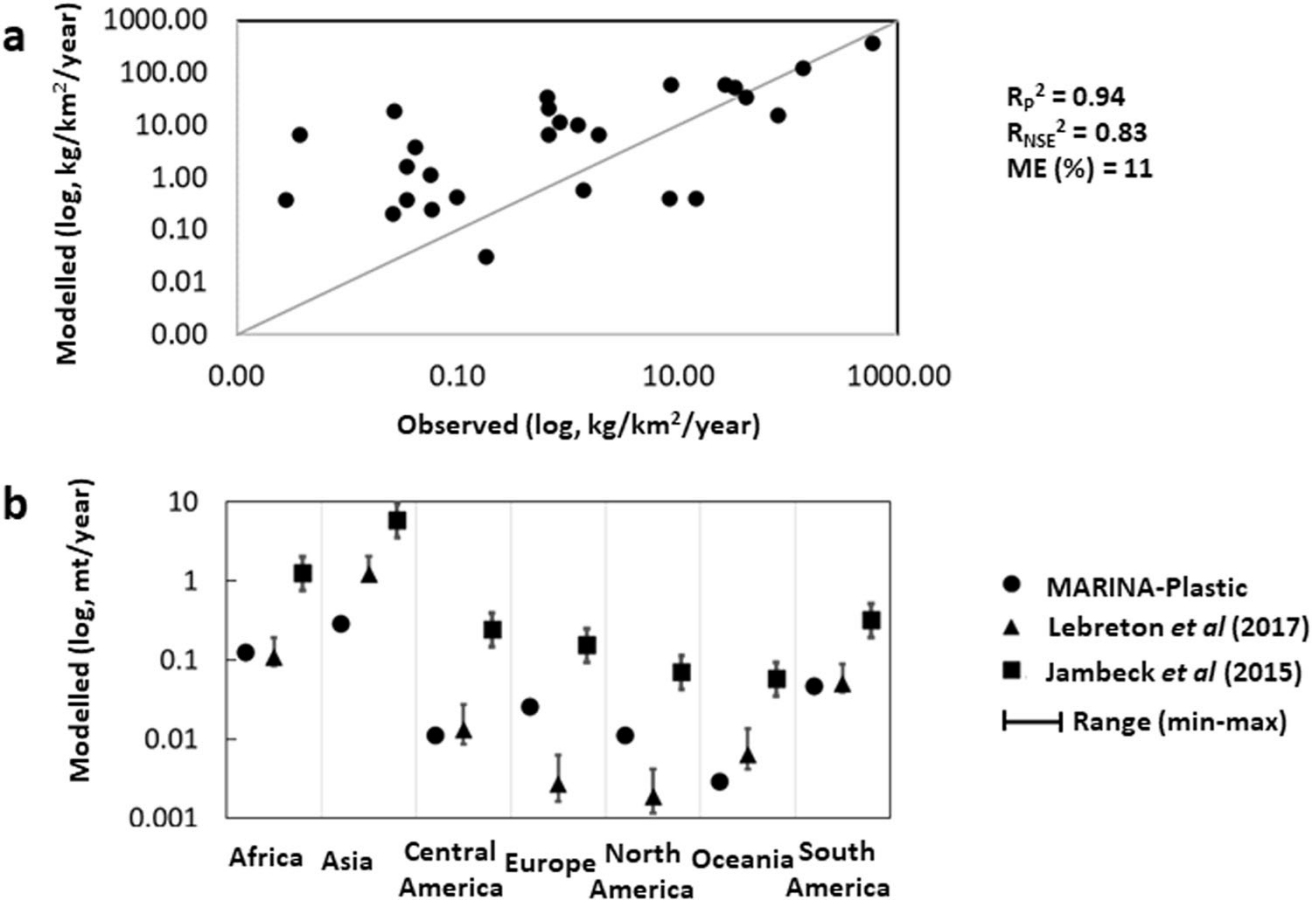
Evaluation of the MARINA-Plastics model for individual rivers. **a** Model validation. **b** Comparison with other models for regions. We compare our modelled values with available observations for individual rivers (see Supplementary Table 7 and Supplementary Fig. 4) and with the models of Lebreton et al.^9^ and Jambeck et al.^8^ for regions (see Supplementary Tables 8, 9). *R*_P_^2^, *R*_NSE_^2^ and ME are the Pearson’s coefficient of determination (fraction, 0–1), the Nash–Sutcliffe efficiency (fraction, 0–1) and the Model Error (%), respectively. *R*_P_^2^ indicates the proportion of the variance in observations that can be explained by the model. *R*_NSE_^2^ shows how well the observed and modelled values fit the line of 1:1. ME indicates the difference between the observed and modelled values. These statistical indicators are calculated according to Moriasi et al.^80^.

Our comparison of model results with observations for 25 rivers indicates an acceptable model performance based on an *R*^2^ of 0.94, *R*_NSE_^2^ of 0.83, and Model Error of 11% (on a log scale, Fig. 6, see the “Methods” section for more details on model evaluation). Our comparison with other studies shows the following. For microplastics, our annual global riverine estimate of 53 kton is much higher than 6.1–6.6 kton from Weiss et al.^35^. (when we look only at *R*^2^ of 0.80 in their study), slightly higher than 47 kton from van Wijnen et al.^14^, much lower than 236 kton from Van Sebille et al.^34^, but within the range of 35–66 kton from Eriksen et al.^36^ For macroplastics, our global annual riverine estimate of 0.5 million tons is at the higher corner of 0.15–0.53 million tons from Mai et al.^37^, slightly higher than 0.4 million tons from Schmidt et al.^26^, higher than 0.1 million tons from Mai et al.^3^, within the range of 0.3–1.5 million ton from Nakayama and Osako^29^, lower than 0.70 million tons from Zhang et al.^38^ and the estimates of 1.1–22 million tons from the other studies (see Supplementary Table 8 for references). For Europe (around 20 kton) and North America (around 4 kton), our river export of macroplastics (Fig. 1) is between the estimates of Lebreton et al.^9^ and Jambeck et al.^8^ (Fig. 6). For Africa (around 120 kton), Central (10 kton) and South America (around 40 kton), our macroplastics are comparable with Lebreton et al.^9^, but for Asia (around 350 kton) and Oceania (2 kton), we are generally lower than in Lebreton et al.^9^ and Jambeck et al.^8^ (Figs. 1 and 6). Our hotspots of riverine plastic exports to coastal waters match the hotspots of the other studies^10,14,26,29^.

The differences in the estimates between our and existing studies are associated with different modelling approaches, data sources, and with time and space (Supplementary Table 9). For example, Weiss et al.^35^ collected existing datasets and estimated microplastics in seas using different factors to convert particles to mass. Their global estimates vary from 6.1 to 6.6 kton under *R*^2^ of 0.80 and from 3.5 to 4610 kton under *R*^2^ of 0.45–0.69. It indicates the importance of the conversions to mass in the approach of Weiss et al.^35^. In our model, we do not convert, but directly estimate the mass of microplastics and use leakage rates to estimate the fragmentation of macroplastics into microplastics. These leakage rates depend on human developments and vary among sub-basins (see “Methods”, Supplementary Tables 4 and 5, Supplementary Fig. 3), which is different from van Wijnen et al.^14^, who used a fixed value of 50%. In general, our process-based modelling approach is comparable to the approach of van Wijnen et al.^14^ and Siegfried et al.^20^ for microplastics, but differs from the approaches of Jambeck et al.^8^ Lebreton et al.^9^, Mai et al.^3^ (empirically-based), Meijer et al.^13^ (probability-based) and Jang et al.^28^ (based on stock and flow) for macroplastics (Supplementary Table 9). The model by Jambeck et al.^8^ did not consider exclusively rivers but, more generally, coastal regions as plastic sources. They estimated the land-based plastics entering oceans using the relation with economic activities, population and solid waste. Lebreton et al.^9^ focused on the riverine export of plastics using hydrological information, solid waste management and population. Other models take mismanaged waste as a predictor for plastic flux^26^ or consider the cycle of plastics in the marine environment^31^. Our modelling approach differs in scale (sub-basin) and sources (point and diffuse) from those models (details are in Supplementary Table 9).

### Our contributions to knowledge gaps for pollution reduction

We developed the MARINA-Plastics model that enables us to increase our understanding in three ways. First, we better understand the interrelations between macro- and microplastics because we consider the fragmentation of macroplastics into microplastics (Knowledge gap 1). This means that changing macroplastics will also affect microplastics in rivers. This insight is relevant for pollution reduction. Second, we provide sub-basin scale analyses to better understand the origin of plastics in seas (Knowledge gap 2, Fig. 2). We show which sub-basins are dominated by point and/or diffuse sources (Knowledge gap 3, Figs. 3 and 4). These insights help to identify sub-basins that need actions today (highly polluted) and develop strategies that target pollution sources: e.g., point sources in Europe and North America and diffuse sources in Africa, Asia and South America (Figs. 2 and 3). Third, our classification of sub-basins integrates information on the shares of macro- and microplastics and their sources (Knowledge gap 4). We show that almost 40% of the global sub-basins are predominantly polluted with microplastics from point sources (sewage systems), but approximately 80% of the global population live in sub-basins that are predominantly polluted with macroplastics from diffuse sources (mismanaged solid waste). In 10% of the global sub-basins, point and diffuse sources are important contributors to plastic pollution (Figs. 2–4).

Our study may help to prioritise pollution reduction strategies for point sources (Classes I and III sub-basins) and diffuse sources (Classes II and III sub-basins) to reduce sea pollution worldwide. Strategies for point sources could be reduced consumption of products such as personal care products^39^ and improved wastewater treatment^40–42^ to avoid microplastics in rivers from sewage systems. Strategies for diffuse sources could be a better collection of solid waste and a ban on single-use plastic products to avoid macroplastics in rivers from mismanaged waste. Both macro- and microplastic should be considered when designing strategies. However, in the real world, resources are limited. It is therefore important to determine which plastic (macro- and/or micro) is most abundant in a basin and from which source to make the first step to prioritise the policy focus. For example, for Europe and North America, the priority could be to reduce point sources. For Asia and Africa, the priority could be to reduce diffuse sources because, in these regions, solid waste collections are not well managed, but the production of such waste is large^43^. Furthermore, solid waste from industrialised countries (e.g., European countries located in Class I sub-basins) can also be exported to less developed countries in Asia (located in Class II sub-basins), contributing to the production of mismanaged plastic waste^43^. Thus, reducing mismanaged waste in industrialised countries could potentially reduce also pollution in Asian countries. The efforts to reduce plastic pollution could help to achieve SDGs^44^. For example, reducing plastic pollution from rivers will directly support SDG6 “clean water and sanitation” and 14 “life below water”. Supporting other SDGs will depend on reduction options: e.g., improved wastewater treatment^23,45^ will contribute to SDG11 “sustainable cities”, and reduced mismanaged plastic waste^2,46^ will contribute to SDG 12 “sustainable consumption” (Supplementary Fig. 6).

### Uncertainties

Uncertainties are generally large in global models. In our plastic model, the estimates vary by up to four orders of magnitude^47^ (see “Comparisons with other studies”). In our study, uncertainties are largely associated with data processing (Supplementary Table 5), model parameters (Supplementary Table 4), and the modelling approach (Supplementary Discussion). Not all data were available at the sub-basin scale^32^. Some data were available at the grid (e.g., mismanaged solid waste)^2^ and national scales (e.g., sewage connections, human development index)^2,32,48,49^. For processing data, we often used the gridded population as commonly done^50,51^ (see more examples in Supplementary Discussion). Several model parameters are uncertain including leakage rates that reflect the relationship between macro- and microplastics^35^ and the per capita input of microplastic from car tyre wear in sewage systems^52^. For the leakage rate, we used expert knowledge supported by literature (Supplementary Tables 4–6). This may differ for other models. For example, Weiss et al.^35^ showed how different techniques to convert items to mass may result in the variation of microplastic fluxes by several orders of magnitude (see examples in the “Comparisons with other studies” section above). Our model does not need these conversion factors, but it is simplified for the accumulation of plastics in rivers. For car tyre wear, we used the approach of Siegfried et al.^20^, which was adopted for the global scale by Strokal et al.^32^ (see “Methods”). We realise that the amount and size of released wear particles from the tyre depend on many factors such as temperature, the structure of the tyre and roads^53,54^. Car tyre wear particles consist of 40–60% of rubber content (synthetic and natural), 20–35% of filler (carbon black and silica) and 12–15% of oil^52,55^. Car tyre wear is considered as an important source of microplastics in soils, air and sewage^52,54,56–58^. This is because microplastics are generally characterised as polymer-based materials, which is similar to the description of the car tyre wear particles according to literature^52,55^. In our study, we only consider microplastics entering sewage from car tyre wear (see “Methods” and Supplementary Table 4). We do not consider wear-associated microplastics in soils and air. Thus, our microplastic pollution levels in rivers might be underestimated. On the other hand, consisting only partially of synthetic polymers, the amount of microplastics from tyre wear may be overestimated.

Rivers can serve as sinks^15^, leading to lower riverine exports. In contrast, floods can increase plastic mobilisation^29,59^ and overflow of sewage, leading to higher riverine exports^59^. In our model, retention rates of plastics are annual and are considered through two factors: (1) water consumption, and (2) sedimentation and beaching (see Eq. 4 in “Methods”). It is a lumped, simple approach that saves the commutation time and allows estimating annual plastic fluxes for data-poor sub-basins (e.g., in Africa), but this approach may underestimate plastic accumulation and thus overestimate riverine plastic export. Our model also ignores plastics in rivers from agricultural films^60^, industries^6^, ships^61^ and deposited from the air^62^, leading to the underestimation of our riverine exports. Nevertheless, we believe that we account for the most relevant sources of macro- and microplastics associated with urbanisation and waste management, which is in line with other studies^2,31,63,64^.

To better understand the impact of uncertainties, we performed a sensitivity analysis (Supplementary Table 10 and Supplementary Fig. 5). We selected 16 model inputs reflecting the calculations of the point- and diffuse sources of plastics to rivers and their river exports (see the list in “Methods” and Supplementary Table 10). We altered those inputs by +10% and −10%. Our results indicate a low sensitivity of the model outputs to changes in most inputs including the per capita input of microplastics in sewage from car tyre wear, personal care products, household dust, and laundry (Supplementary Fig. 5). River export of microplastics is somewhat sensitive (0–30%) to changes in wastewater treatment removals (model input 6 in Supplementary Fig. 5). For river export of macroplastics, this holds for changes in mismanaged solid waste and leakage rates (model inputs 8 and 9 in Supplementary Fig. 5). A similar conclusion is for retention rates of plastics in rivers (model inputs 13 and 15 in Supplementary Fig. 5). Considering these results, we believe that uncertainties in model inputs do not largely affect our messages on a global and regional scale. However, for local analysis of plastic pollution, the model needs to be further validated and checked for specific local conditions.

In summary, our model estimates that approximately 0.5 million tons of plastics reach the seas from rivers each year worldwide. Most of these plastics are macroplastics in terms of mass. However, there is a large spatial variability in the riverine plastic exports and their sources among sub-basins. In this study, we show that almost 40% of the global sub-basins in our model are dominated by microplastic pollution from point sources (sewage systems). These sub-basins are located in Europe, North America and Oceania. Approximately 80% of the global population lives in river sub-basins where plastic export to seas is dominated by macroplastics from diffuse sources (mismanaged waste). These sub-basins are located in Asia and Africa. In 10% of the global sub-basins, rivers export the dominant amounts of both macro- and microplastics from point and diffuse sources. These sub-basins are located in parts of Europe, North and South America. These insights could be useful to prioritise reduction strategies to avoid future plastic pollution in seas worldwide.

## Methods

We developed the Model to Assess River Inputs of pollutaNts to seAs for plastics (MARINA-Plastics model) to quantify annual river export of macro- and microplastics by source and sub-basin (Supplementary Fig. 1). Sub-basins are defined as sub-catchments that cover the surface area of the world (excluding oceans). The term “sub-basin” is used in the other versions of the MARINA models^32,65,66^. MARINA-Plastics was developed based on an existing MARINA-Multi model^32^. The existing MARINA-Multi model quantifies annual inputs of multiple pollutants to rivers from sewage systems for 10,226 sub-basins^32^. These sub-basins were delineated in an earlier study^67^ using the water flow and hydrology from the Variable Infiltration Capacity (VIC) model^68^. The MARINA-Multi model estimates riverine transport of nutrients, a pathogen, a chemical (triclosan) and microplastics. The current study further develops the microplastic component of the MARINA-Multi model (Supplementary Fig. 1). These improvements include three main aspects. First, we developed a modelling approach to quantify annual inputs of macroplastics from mismanaged solid waste to rivers. We did this by studying mass-balance approaches for other pollutants^66,69,70^ that inspired us to develop an approach for macroplastics. Second, we added a source of microplastics in rivers from macroplastic degradation. We used the approach of van Wijnen et al.^14^ at the sub-basin scale for river exports of plastics. Third, we adjusted and integrated the sub-basin scale modelling approach^32,69,71^ to quantify river export of macro- and microplastics by source from sub-basins (see details below). The sub-basin scale modelling was developed for river export of nutrients in China^65,69^, but had never been applied for river exports of macro- and microplastics worldwide. River exports of macro- and microplastics are calculated as a function of hydrology, retention and removal rates of macro- and microplastics in rivers and their export fractions towards the sub-basin outlets and river mouths. All these three developments resulted in the MARINA-Plastics model that is used in this study.

The model quantifies river export of plastics to seas from sub-basins and sources as a function of socio-economic developments, urbanisation, waste management and hydrology (Eq. (1)). This is done by correcting the inputs of plastics to rivers with the retention rates in the river systems (e.g., along the river banks, fragmentations, water consumption, Eqs. (1), (2) and (6)). In general, two steps are included to calculate river exports of plastics. The first step is to calculate the inputs of plastics to rivers. For microplastics, we have inputs from diffuse and point sources. Diffuse source inputs are the release of microplastics from macroplastics in rivers. Point source inputs are sewage systems discharging microplastics from car tyres, laundry, personal care products and household dust. These point source inputs only depend on the removal efficiencies during treatment. For macroplastics, we have diffuse source inputs, which are mismanaged plastic waste entering rivers via, for example, surface runoff. *The second step* is to calculate the retention of plastics in rivers to calculate river exports. Retention rates include the loss of plastics from rivers via water consumption and retention rates in rivers as a result of, for example, sedimentation.

Our model provides outputs for sub-basins (Supplementary Tables 1 and 2). We define two categories of sub-basins based on Strokal et al.^32^. The first category is for 29 large river basins (Supplementary Table 3). Their drainage areas are divided into smaller up-, middle- and downstream sub-basins. The model calculates river exports of those up-, middle- and downstream sub-basins. We have the model outputs for each sub-basin. The total river export of plastics from all sub-basins is the sum of river exports of up-, middle- and downstream sub-basins. The second category consists of the sub-basins that are individual and drain directly to the river mouth. This implies that these sub-basins are considered downstream sub-basins.

Below, we describe the equations to quantify river export of macro- (Eqs. (1), (2)–(5)) and microplastics (Eqs. (1), (6)–(16)).

### Quantifying the total river export of plastics to seas by source

This is done as a function of river export of macro- and microplastics. River export of macroplastics is calculated as a function of mismanaged waste production and retention in soils and in river systems. River export of microplastics is calculated as a function of the population with sewage connections, wastewater treatment efficiencies, per capita microplastic production (e.g., via car tyre wear) or consumption (e.g., via personal care products) rates and retention rates in river systems. Equations and associated details are given below.

River export of plastics from sub-basins is calculated as follows:

1 $${{Ld}}_{{plastic}.j}=\,{{Ld}}_{{mi}.j}+\,{{Ld}}_{{ma}.j}$$

Where *Ld*_*plastic.j*_ is the total annual plastic export by rivers to sea from sub-basin j from all sources (kg/year); *Ld*_*mi.j*_ is the total annual microplastic export by rivers to sea from sub-basin *j* from all sources (kg/year); *Ld*_*ma.j*_ is the total annual macroplastic export by rivers to sea from sub-basin *j* from all sources (kg/year).

### Quantifying river export of macroplastics by source

River export of macroplastics by source from sub-basins is quantified based on our modelling approach but integrated into the sub-basin-scale modelling^32,69^:

2 $${{Ld}}_{{ma}.j}=\left({{WS}}_{j}-\,{{RS}}_{{diff}.{mi}.j}\right)\times {{FE}}_{{riv}.{ma}.o.j}\times {{FE}}_{{riv}.{ma}.m.j}$$

...where, ${{WS}}_{j}$ is the input of mismanaged macroplastic waste in sub-basin *j* (kg/year); ${{RS}}_{{diff}.{mi}.j}$ is the diffuse-source input of microplastics to rivers as a result of fragmentation of macroplastics in sub-basin *j* (kg/year, see next section); ${{FE}}_{{riv}.{ma}.o.j}$ is the fraction of macroplastics in rivers that are exported to the outlet of sub-basin *j* (fraction, 0–1). This includes retention, such as along the river banks, fragmentations and water consumption; ${{FE}}_{{riv}.{ma}.m.j}$ is the fraction of macroplastics that are exported from the outlet of sub-basin j to the river mouth (fraction, 0–1). The full description of the calculation is given in Supplementary Tables 1–3, Supplementary Fig. 2.

3 $${{WS}}_{j}={P}_{{MPW}.j}\times {F}_{{leakage},j}$$

...where, ${P}_{{MPW}.{j}}$ is the production of mismanaged macroplastic waste in sub-basin *j* (kg/year); ${F}_{{leakage},j}$ is the fraction of macroplastics reaching rivers from mismanaged macroplastic waste in sub-basin *j* (fraction, 0–1).

4 $${{FE}}_{{riv}.{ma}.o.j}=(1-{L}_{{ma}.j})\times (1-{{FQ}}_{{rem}.j})$$

...where, ${L}_{{ma}.j}$ is the combined retention factor of macroplastics in rivers as a result of sedimentation and beaching (fraction, 0-1fraction, fraction, 0-1), based on research^72^; ${{FQ}}_{{rem}.j}$ is the fraction of water that is removed from the river system for different purposes (e.g., irrigation) in sub-basin j (fraction, 0–1).

5 $${{FQ}}_{{rem}.j}=\,1-\frac{{{Qact}}_{j}}{{{Qnat}}_{j}}$$

...where, ${{Qact}}_{j}$ is the actual river discharges at the outlet of sub-basin *j* after correcting for water removal (km^3^/year); ${{Qnat}}_{j}$ is the natural river discharges at the outlet of sub-basin *j* without correcting for water removal (km^3^/year).

### Quantifying river export of microplastics by source

River export of microplastics by source from sub-basins is quantified based on adjusted modelling approaches of^14,32,71^:

6 $${{Ld}}_{{mi}.j}=\left({{RS}}_{{diff}.{mi}.j}+\,{{RS}}_{{pnt}.{mi}.j}\right)\times \,{{FE}}_{{riv}.{mi}.o.j}\times \,{{FE}}_{{riv}.{mi}.m.j}$$

...where, ${{RS}}_{{diff}.{mi}.j}$ is the diffuse-source input of microplastics to rivers as a result of fragmentation of macroplastics in sub-basin *j* (kg/year); ${{RS}}_{{pnt}.{mi}.j}$ is the point-source input of microplastics to rivers from sewage systems in sub-basin *j* (kg/year); ${{FE}}_{{riv}.{mi}.o.j}$ is the fraction of microplastics in rivers that are exported to the outlet of sub-basin *j* (fraction, 0–1). This includes retention along the river banks, fragmentations and water consumption; ${{FE}}_{{riv}.{mi}.m.j}$ is the fraction of microplastics that are exported from the outlet of sub-basin *j* to the river mouth (fraction, 0–1). The full description of the calculation is given in Supplementary Tables 1–3, Supplementary Fig. 2.

7 $${{RS}}_{{diff}.{mi}.j}=\left({{WS}}_{f.j}\,\times \,{t}_{{res}.f.j}+\,{{WS}}_{s.j}\,\times \,{t}_{{res}.s}\right)\times \,{F}_{{ma}}$$

...where, ${{WS}}_{f.j}$ is the input of macroplastics into the fast fraction in sub-basin *j* (kg/year); ${{WS}}_{s.j}$ is the input of macroplastics into the slow fraction in sub-basin *j* (kg/year); ${t}_{{res}.f.j}$ is the average residence time of macroplastics in the fast fraction in sub-basin *j* (year). It is calculated using van the approach of Wijnen et al.^73^ but adjusted to sub-basins; ${t}_{{res}.s}$ is the average residence time of macroplastics in the slow fraction (year); ${F}_{{ma}}$ is the relative release rate of microplastics from macroplastics (/year).

8 $${{WS}}_{f.j}={{FR}}_{f}\,\times \,{{WS}}_{j}$$

...where, ${{FR}}_{f}$ is the relative share of ${{WS}}_{j}$ in the fast fraction (fraction, 0–1); ${{WS}}_{j}$ is the input of mismanaged macroplastic waste in sub-basin *j* (kg/year).

9 $${{WS}}_{s.j}={{FR}}_{s}\,\times \,{{WS}}_{j}$$

...where, ${{FR}}_{s}$ is the relative share of ${{WS}}_{j}$ in the slow fraction (fraction, 0–1).

10a $${t}_{{res}.f.j}=\frac{{{Area}}_{{land}.j}}{{{Area}}_{{average}}\times 60}\times \frac{1}{365}$$

If sub-basins drain directly into the coastal waters and/or the land area of sub-basins is larger than 5000 km^2^, then ${t}_{{res}.f.j}$ is calculated using Eq. (10b) instead of Eq. (10a) according to van Wijnen et al.^14^:

10b $${t}_{{res}.f.j}=\left(0.4+0.6\,\times \,\frac{5000}{{{Area}}_{{land}}}\right)\times \left(\frac{{{Area}}_{{land}.j}}{{{Area}}_{{average}}\times 60}\times \frac{1}{365}\right)$$

...where, ${{Area}}_{{land},j}$ is the total land area of sub-basin *j* (km^2^); ${{Area}}_{{average}}$ is the average land area of the 50 largest river basins in the world (km^2^)

11 $${{RS}}_{{pnt}.{mi}.j}={{WSdif}}_{{mi}.j}\times \left(1-{{hw}}_{{mi}.j}\right)\times {{PopCon}}_{j}$$

...where, ${{WSdif}}_{{mi}.j}$ is the consumption or production rate of microplastics in sub-basin *j* (kg/cap/year); ${{hw}}_{{mi}.j}$ is the removal fraction of microplastics in sub-basin *j* (fraction, 0–1); ${{PopCon}}_{j}$ is the total population connected to sewage systems in sub-basin *j* (people/year).

12 $${{WSdif}}_{{mi}.j}={{WSdif}}_{{laundry}.j}+{{WSdif}}_{{tyres}.j}+{{WSdif}}_{{pcp}.j}+{{WSdif}}_{{dust}.j}$$

...where, ${{WSdif}}_{{laundry}.j}$ is the microplastic rate from laundry in sub-basin *j* (kg/cap/year); ${{WSdif}}_{{tyres}.j}$ is the microplastic rate from car tyres in sub-basin *j* (kg/cap/year); ${{WSdif}}_{{pcp}.j}$ is the microplastic rate from personal care products in sub-basin *j* (kg/cap/year); ${{WSdif}}_{{dust}.j}$ is the microplastic rate from household dust in sub-basin *j* (kg/cap/year).

13 $${{PopCon}}_{j}={{UrbCon}}_{{{{\rm{j}}}}}+{{RurCon}}_{{{{\rm{j}}}}}$$

...where, ${{UrbCon}}_{{{{\rm{j}}}}}$ is the urban population connected to sewage systems in sub-basin *j* (people/year); ${{RurCon}}_{{{{\rm{j}}}}}$ is the rural population connected to sewage systems in sub-basin *j* (people/year).

14 $${{UrbCon}}_{{{{\rm{j}}}}}={{Urb}}_{{{{\rm{j}}}}}\,\cdot \,{{fr}}_{{{{\rm{urb}}}}.{{{\rm{con}}}}.{{{\rm{j}}}}}$$

...where, ${{Urb}}_{{{{\rm{j}}}}}$ is the urban population in sub-basin *j* (people/year); ${{fr}}_{{{{\rm{urb}}}}.{{{\rm{con}}}}.{{{\rm{j}}}}}$ is the fraction of the urban population connected to sewage systems in sub-basin *j* (fraction, 0–1).

15 $${{RurCon}}_{{{{\rm{j}}}}}={{Rur}}_{{{{\rm{j}}}}}\,\cdot \,{{fr}}_{{{{\rm{rur}}}}.{{{\rm{con}}}}.{{{\rm{j}}}}}$$

...where, ${{Rur}}_{{{{\rm{j}}}}}$ is the rural population in sub-basin *j* (people/year); ${{fr}}_{{{{\rm{rur}}}}.{{{\rm{con}}}}.{{{\rm{j}}}}}$ is the fraction of the rural population connected to sewage systems in sub-basin *j* (fraction, 0–1).

16 $${{FE}}_{{riv}.{mi}.o.j}=(1-{L}_{{mi}.j})\times (1-{{FQ}}_{{rem}.j})$$

...where, ${L}_{{mi}.j}$ is the retention factor of microplastics in rivers as a result of sedimentation in sub-basin *j* (fraction, 0–1).

### Data sources and processing

The model inputs include population connected to sewage systems, removal fractions of microplastics during treatment, per capita consumption or production rates of microplastics from personal care products, laundry, household dust and car tyre wear. All these inputs are available in Strokal et al.^32^ at the grid scale of 0.5°, and these gridded inputs are aggregated to sub-basins for this study (see details in Supplementary Tables 4 and 5). The data of Strokal et al.^32^ are collected from other existing datasets and models^14,20^. The data of Strokal et al.^32^ are freely published in Strokal et al.^74^ Supplementary Tables 4–6 provide details on the data sources and how data are processed in our study. It is important to note that the per capita input of microplastics to sewage systems from car tyre wear is based on the approach of Siegfried et al.^20^ that was adopted by Strokal et al.^32^. Only a part of a tyre wear particle consists of microplastics. Therefore, corrections have been made to the approach of Siegfried et al.^20^. Large-scale modelling studies by Siegfried et al.^20^ and van Wijnen et al.^14^ looked at car tyre wear that can enter sewage systems (one-third of the total). These two modelling studies estimated the per capita emission of microplastic from car tyre wear considering car numbers and car tyre wear production in Europe (18 kg/cap/year). Siegfried et al.^20^. applied 0.18 kg/cap/year for the European basins to quantify river export of microplastics from sewage-related car tyre wear. Strokal et al.^32^ adopted this method for the global scale to quantify inputs of microplastics from sewage-related car tyre wear. In our study, we applied the approach of Strokal et al.^32^ (see Supplementary Table 4 for the data).

Model inputs for calculating macroplastics in rivers include mismanaged plastic waste and leakage rates. Mismanaged plastic waste is available in Lebreton and Andrady^2^. These data are processed into sub-basins as explained in Supplementary Table 5. The leakage rates are derived from literature^75–77^ and supported by characteristics of sub-basins for human development (see justifications in Supplementary Table 5). Macroplastics are also a source of microplastics in rivers (see Eqs. (6)–(9)). To calculate microplastics from macroplastics, several model inputs are needed and derived from existing studies^14,20^ (see sources in Supplementary Table 4).

Model inputs for calculating river exports of macro- and microplastics include river discharges (for *FQrem*_*j*_), retention fractions of macro- and microplastics in rivers as a result of degradation (*L*_*mi.j*_ and *L*_*ma.j*_), and areas with the main channel in sub-basins (for *FE*_*riv.mi.m.j*_ and *FE*_*riv.ma.m.j*_, Supplementary Tables 1–3). The model distinguishes the main channel and tributaries. Tributaries export plastics to the main channel, and the main channel exports plastics to the river mouth. Thus, the model has sub-basins with the main channel and tributaries. This is needed to define the routing scheme (see Supplementary Tables 1 and 2). Every sub-basin has an outlet in the model, which is the point of plastic arrival from, for example, tributaries. The model distinguishes natural (without human influences) and actual (with human influences such as irrigation) river discharges. Natural river discharges are derived from a hydrological model^48,49^ at the grid of 0.5° where the sub-basin outlets are located. Actual river discharges are estimated using the ratio between natural and actual river discharges from Fekete et al.^78^. Details are in Supplementary Tables 4, 5. Retention rates of microplastics in rivers are based on Siegfried et al.^20^. Macroplastics retention rates are estimated using data from Schöneich-Argent et al.^72^. The retention factor is based on the ratio of exported macroplastics to coastal waters versus accumulated macroplastics in the rivers (adapted from Schöneich-Argent et al.^72^). Supplementary Table 6 provides details on the data used to estimate the retention rates.

### Model evaluation and sensitivity analysis

Our model is based on the earlier point-source version (MARINA-Multi), which has been evaluated^32^. In this study, we further evaluated the MARINA-Plastics model in three ways^32^ following a “building trust circle” approach. This approach was developed for large-scale water quality models for which observations are limited^32,79^. The approach has been applied in evaluating global models^66^ and includes six ways. In this study, we follow three out of six ways: (1) validation, (2) comparisons with other studies and (3) sensitivity analysis.

First, we validated the model using the available observations. Supplementary Table 7 provides collected observations for macroplastic export to coastal waters from 15 studies for 25 rivers that are mainly located in Europe. The locations of these rivers are shown in Supplementary Fig. 4. We plotted our modelled values for river export of macroplastics with observed values on the 1:1 line. We calculated the three statistical indicators: *R*_P_^2^, *R*_NSE_^2^ and ME, according to Moriasi et al.^80^. *R*_P_^2^ is Pearson’s coefficient of determination and shows the proportion of the variance in observed values that can be explained by the model. R_P_^2^ ranges from 0 to 1. Closer to 1 indicates a better model performance. *R*_NSE_^2^ is the Nash-Sutcliffe efficiency and indicates how well-modelled and observed values fit on the 1:1 line (fraction, 0–1). Values above 0.5 generally indicate a good model performance^80^. ME is the Model Error (%), which is the difference between modelled and observed values. Results are shown in Fig. 5 and discussed in the “Results and discussion” section of the main text. It has to be mentioned that our observations are for a set of individual rivers. Thus, the results of the statistical indicators have to be considered with caution and in combination with the results of the other two ways that are used to evaluate the model (see below).

Second, we compared our model results with other studies (see the “Results and discussion” section). We collected global and regional estimates from existing models. Supplementary Table 8 provides global comparisons and Fig. 5 provides regional comparisons. In addition, we also compared our modelling approaches with other models (Supplementary Table 9) and model results for individual rivers (see the “Results and discussion” section for the references).

Third, we performed a sensitivity analysis. We changed 16 model inputs by +10%. Supplementary Table 10 shows the set-up of the sensitivity analysis. The chosen 16 model inputs are:

1. The fraction of the urban population connected to sewage (${{fr}}_{{{{\rm{urb}}}}.{{{\rm{con}}}}.{{{\rm{j}}}}}$, fraction, 0–1, Eq. (14));
2. The consumption rate of microplastics from car tyres (${{WSdif}}_{{tyres}.j}$, kg/cap/year, Eq. (12));
3. The consumption rate of microplastics from PCP (${{WSdif}}_{{pcp}.j}$, kg/cap/year, Eq. (12));
4. The consumption rate of microplastics from dust (${{WSdif}}_{{dust}.j}$, kg/cap/year, Eq. (12));
5. The consumption rate of microplastics from laundry (${{WSdif}}_{{laundry}.j}$, kg/cap/year, Eq. (12));
6. The removal fraction of microplastics during treatment (${{hw}}_{{mi}.j}$, fraction, 0–1, Eq. (11));
7. The average area of the largest 50 rivers in the world (${{Area}}_{{average}}$, km^2^, Eqs. (10a) and (10b));
8. Mismanaged plastic waste production (${P}_{{MPW}.j}$, kg/year, Eq. (3));
9. The leakage rate for macroplastics (${F}_{{leakage},j}$, fraction, 0–1, Eq. (3));
10. The release rate of microplastics from macroplastics (${F}_{{ma}}$, /year, Eq. (7));
11. The fast fraction (${t}_{{res}.f.j}$, year, Eq. (7));
12. The slow fraction (${t}_{{res}.s}$, year, Eq. (7));
13. The export fraction of microplastics to the sub-basin outlet (${{FE}}_{{riv}.{mi}.o.j}$, fraction, 0–1, Eq. (6));
14. The export fraction of microplastics to the river mouth (${{FE}}_{{riv}.{mi}.m.j}$, fraction, 0–1, Eq. (6));
15. The export fraction of macroplastics to the sub-basin outlet (${{FE}}_{{riv}.{ma}.o.j}$, fraction, 0–1, Eq. (2));
16. The export fraction of macroplastics to the river mouth (${{FE}}_{{riv}.{ma}.m.j}$, fraction, 0–1, Eq. (2)).

The choice for these inputs is justified by their influence on the calculations of the point- and diffuse-source inputs of macro- and microplastics to rivers and their exports to the river mouth (coastal waters, see the equations above). Some of these model inputs are generic for all sub-basins (e.g., $\,{{WSdif}}_{{pcp}.j}$, ${{WSdif}}_{{pcp}.j}$, ${{WSdif}}_{{dust}.j}$, ${{WSdif}}_{{laundry}.j}$, ${t}_{{res}.s}$, Supplementary Table 4). The sensitivity analysis is used to better understand how uncertainties in these model inputs influence model outputs. Results of the sensitivity analysis are shown in Supplementary Fig. 5 and discussed in the “Results and discussion” section of the main text.

### Three classes for river exports of plastics

We classify sub-basins according to three classes for river exports of plastics (see also Supplementary Note): I–III. Class I sub-basins are dominated by microplastic pollution in rivers, and over 70% of this amount is from point sources (sewage systems). These are the sub-basins in which rivers export over 70% of plastics as microplastics. Class II sub-basins are dominated by macroplastic pollution in rivers, and over 70% of this amount is from diffuse sources (mismanaged solid waste). These are the sub-basins in which rivers export over 70% of plastics as macroplastics. Class III sub-basins include rivers where macro- and microplastic have a more equal share (30–70%) in the total plastic export. Point and diffuse sources are important contributors. This indicates that in sub-basins under Class III, both macro- and microplastics can be important.

We present the analysis for each class in Fig. 5 of the main text. In this figure, we focus on the share of sub-basin areas, sewerage connections, treatment rates, and mismanaged plastic waste. The sewage connection (%) reflects the average situation in each region (each region consists of the sub-basins, see Supplementary Fig. 2b for the definition of the regions). For each region, the average sewage connection (%) is calculated as follows: the total population of the region with the sewage connection is divided by the total population of the region and then multiplied by 100 to get a percentage. The same method was applied to calculate the average treatment rates for regions. For mismanaged plastic waste, we summed mismanaged plastic waste in kg/year over the sub-basins and divided it by the total area of those sub-basins in the region.

### Supplementary information

Supplementary information

## Data Availability

All data and materials are available in the Supporting Information to this manuscript. In addition, The main model results supporting Figs. 1–5 generated in this study have been deposited in the DANS Easy repository under the accession code 10.17026/dans-xaa-kug9. The other data that underline the estimates of the model results are presented in the supporting information with references.

## Code availability

All equations to the model are provided in the “Methods” section.
