# Supplementary material for: River export of macro- and microplastics to seas by sources worldwide
Source: Nat Commun. 2023 Aug 10;14:4842. doi: 10.1038/s41467-023-40501-9 (PMC10415377; doi:10.1038/s41467-023-40501-9)
Supplement: Supplementary file 1 — Supplementary information [file 41467_2023_40501_MOESM1_ESM.pdf]

Supplementary information

River export of macro- and microplastics to seas by sources worldwide

Maryna Strokal[1*], Paul Vriend[2*], Mirjam P. Bak[1], Carolien Kroeze[3], Jikke van Wijnen[4], Tim van Emmerik[5]

[1]Water Systems and Global Change Group, Wageningen University, Wageningen, the Netherlands

[2]Ministry of Infrastructure and Water Management, Directorate-General for Public Works and Water Management, Utrecht, Netherlands;

[3]Environmental Systems Analysis Group, Wageningen University, Wageningen, the Netherlands;

[4]Department of Environmental Sciences, Faculty of Science, Open University, Heerlen, the Netherlands;

[5]Hydrology and Quantitative Water Management Group, Wageningen University, Wageningen, the Netherlands

Supplementary Table 1. **The description of how to calculate $FE_{riv.mi.m.j}$ according to an existing sub-basin modelling approach of [1,2], specifically adjusted to microplastics.** $FE_{riv.mi.m.j}$ is the fraction of microplastics (mi) exported from the outlet of sub-basin (j) to the river mouth. The same equations are applied to macroplastics ($FE_{riv.ma.m.j}$). Sub-basins are defined as sub-catchments that cover the surface area (excluding oceans). $FE_{riv.mi.m.j}$ is calculated for upstream sub-basins with tributaries ($FE_{riv.mi.m.juT}$) and main channel ($FE_{riv.mi.m.juC}$); middlestream sub-basins with tributaries ($FE_{riv.mi.m.jmT}$) and main channel ($FE_{riv.mi.m.jmC}$); downstream sub-basins with tributaries ($FE_{riv.mi.m.jdT}$) and main channel ($FE_{riv.mi.m.jdT}$). Variables are described in Supplementary Table 2. The locations of up-, middle- and downstream sub-basins are in Supplementary Figure 2.

**Sub-basins (j)**	upstream sub-basins (ju)	middlestream sub-basins (jm)	downstream sub-basins (jd)
Sub-basins formed by tributaries (T)	juT	jmT	jdT
Sub-basins with the main channel (C)	juC	jmC	jdC

(A) $FE_{riv.mi.m.juT} = {}^{juT}FE_{riv.mi.o.juC} \cdot {}^{juT}FE_{riv.mi.o.jmC} \cdot {}^{juT}FE_{riv.mi.o.jdC}$

(B) $FE_{riv.mi.m.juC} = {}^{juC}FE_{riv.mi.o.jmC} \cdot {}^{juC}FE_{riv.mi.o.jdC}$

(C) $FE_{riv.mi.m.jmT} = {}^{jmT}FE_{riv.mi.o.jmC} \cdot {}^{jmT}FE_{riv.mi.o.jdC}$

(D) $FE_{riv.mi.m.jmC} = {}^{jmC}FE_{riv.mi.o.jdC}$

(E) $FE_{riv.mi.m.jdT} = {}^{jdT}FE_{riv.mi.o.jdC}$

(F) $FE_{riv.mi.m.jdC} = 1$ (because outlets of down-stream sub-basins discharge directly nutrients to the river mouth)

(A) $FE_{riv.mi.m.juT}$ →

$${}^{juT}FE_{riv.mi.o.juC} = (1-[L_{mi.juC} \cdot {}^{juT}A_{juC}]) \cdot (1-[FQrem_{juC} \cdot {}^{juT}A_{juC}])$$
$${}^{juT}FE_{riv.mi.o.jmC} = (1-[L_{mi.jmC} \cdot {}^{juT}A_{jmC}]) \cdot (1-[FQrem_{jmC} \cdot {}^{juT}A_{jmC}])$$
$${}^{juT}FE_{riv.mi.o.jdC} = (1-[L_{mi.jdC} \cdot {}^{juT}A_{jdC}]) \cdot (1-[FQrem_{jdC} \cdot {}^{juT}A_{jdC}])$$

(B) $FE_{riv.mi.m.juC}$ →

$${}^{juC}FE_{riv.mi.o.jmC} = (1-[L_{mi.jmC} \cdot {}^{juC}A_{jmC}]) \cdot (1-[FQrem_{jmC} \cdot {}^{juC}A_{jmC}])$$
$${}^{juC}FE_{riv.mi.o.jdC} = (1-[L_{mi.jdC} \cdot {}^{juC}A_{jmC}]) \cdot (1-[FQrem_{jdC} \cdot {}^{juC}A_{jdC}])$$

(C) $FE_{riv.mi.m.jmT}$ →

$${}^{jmT}FE_{riv.mi.o.jmC} = (1-[L_{mi.jmC} \cdot {}^{jmT}A_{jmC}]) \cdot (1-[FQrem_{jmC} \cdot {}^{jmT}A_{jmC}])$$
$${}^{jmT}FE_{riv.mi.o.jdC} = (1-[L_{mi.jdC} \cdot {}^{jmT}A_{jdC}]) \cdot (1-[FQrem_{jdC} \cdot {}^{jmT}A_{jdC}])$$

(D) $FE_{riv.mi.m.jmC}$ →
(E) $FE_{riv.mi.m.jdT}$ →

$${}^{jmC}FE_{riv.mi.o.jdC} = (1-[L_{mi.jdC} \cdot {}^{jmC}A_{jdC}]) \cdot (1-[FQrem_{jdC} \cdot {}^{jmC}A_{jdC}])$$

Supplementary Table 2. **The description of the model variables from Supplementary Table 1.** The sub-basin modelling approach of [1,2] is adjusted to microplastics. The adjusted approach is also applied to macroplastics. All units are in the fraction of 0-1. Sub-basins are defined as sub-catchments that cover the surface area (excluding oceans).

Variables	Description
General abbreviations:<br>○ FEriv is export fraction for rivers<br>○ mi is microplastic<br>○ ma is macroplastic<br>○ m is river mouth<br>○ o is sub-basin outlet<br>○ j is sub-basin	
$FE_{riv.mi.m.juT}$	the fraction of microplastics (mi) exported by rivers (riv) from the outlet of upstream tributary (juT) to the river mouth (m)
$FE_{riv.mi.m.juC}$	the fraction of microplastics (mi) exported by rivers (riv) from the outlet of the upstream main channel (juC) to the river mouth (m)
$FE_{riv.mi.m.jmT}$	the fraction of microplastics (mi) exported by rivers (riv) from the outlet of middlestream tributary (jmT) to the river mouth (m)
$FE_{riv.mi.m.jmC}$	the fraction of microplastics (mi) exported by rivers (riv) from the outlet of middlestream main channel (jmC) to the river mouth (m)
$FE_{riv.mi.m.jdT}$	the fraction of microplastics (mi) exported by rivers (riv) from the outlet of downstream tributary (jdT) to the river mouth (m)
$FE_{riv.mi.m.jdC}$	the fraction of microplastics (mi) exported by rivers (riv) from the outlet of downstream main channel (jdC) to the river mouth (m)
${}^{juT}FE_{riv.mi.o.juC}$<br>${}^{juT}FE_{riv.mi.o.jmC}$<br>${}^{juT}FE_{riv.mi.o.jdC}$	fractions of microplastics (mi) exported by rivers (riv) from the outlet of upstream tributary (superscript: juT) to the outlet (o) of the main channel in upstream (subscript: juC), middlestream (subscript: jmC) and downstream (subscript: jdC) sub-basins
${}^{juC}FE_{riv.mi.o.jmC}$<br>${}^{juC}FE_{riv.mi.o.jdC}$	fractions of microplastics (mi) exported by rivers (riv) from the outlet (o) of upstream sub-basins with the main channel (superscript: juC) to the outlet (o) of the main channel in middlestream (subscript: jmC) and downstream (subscript: jdC) sub-basins
${}^{jmT}FE_{riv.mi.o.jmC}$<br>${}^{jmT}FE_{riv.mi.o.jdC}$	fractions of microplastics (mi) exported by rivers (riv) from the outlet (o) of middle tributary (superscript: jmT) to the outlet (o) of the main channel in middlestream (subscript: jmC) and downstream (subscript: jdC) sub-basins
${}^{jmC}FE_{riv.mi.o.jdC}$	the fraction of microplastics (mi) exported by rivers (riv) from the outlet (o) of middlestream sub-basin with the main channel (superscript: jmC) to the outlet (o) of the main channel in downstream (subscript: jdC) sub-basin (the outlet of the this downstream sub-basin is the river mouth)

Supplementary Table 2 continued	
$L_{mi.juC}$<br>$L_{mi.jmC}$<br>$L_{mi.jdC}$	Fractions of microplastics (mi) that are retained in rivers of upstream (juC), middlestream (jmC) and downstream (jdC) sub-basins with the main channel (C)
$FQrem_{juC}$<br>$FQrem_{jmC}$<br>$FQrem_{jdC}$	Fractions of microplastics (generic for all pollutants) that are lost from rivers of upstream (juC), middlestream (jmC) and downstream (jdC) sub-basins with the main channel (C) via water consumption
${}^{juT}A_{juC}$<br>${}^{juT}A_{jmC}$<br>${}^{juT}A_{jdC}$	Drainage area (A) of the main channel (C) in upstream (subscript: juC), middlestream (subscript: jmC) and downstream (subscript: jdC) sub-basins that exports microplastics from the outlet of upstream tributary (superscript: juT). This drainage area was calculated as the fraction to the total sub-basin area. The values are given in Supplementary Table 3
${}^{juC}A_{jmC}$<br>${}^{juC}A_{jdC}$	Drainage area (A) of the main channel (C) in middlestream (subscript: jmC) and downstream (subscript: jdC) sub-basins that exports microplastics from the outlet of upstream main channel (superscript: juC). This drainage area was calculated as the fraction to the total sub-basin area. The values are given in Supplementary Table 3
${}^{jmT}A_{jmC}$<br>${}^{jmT}A_{jdC}$	Drainage area (A) of the main channel (C) in middlestream (subscript: jmC) and downstream (subscript: jdC) sub-basins that exports microplastics from the outlet of middlestream tributary (superscript: jmT). This drainage area was calculated as the fraction to the total sub-basin area. The values are given in Supplementary Table 3
${}^{jmC}A_{jdC}$	Drainage area (A) of the main channel (C) in the downstream (subscript: jdC) sub-basin that exports microplastics from the outlet of middlestream main channel (superscript: jmC). This drainage area was calculated as the fraction to the total sub-basin area. The values are given in Supplementary Table 3

Supplementary Table 3. **The list of the rivers, which drainage areas are divided into sub-basins.** The table shows the area fraction of the main channel relative to the total sub-basin area (0-1). For these sub-basins, the modelling approach of Supplementary Table 1 is applied. The drainage area of the other rivers in the world (see Supplementary Figure 2) is considered as individual sub-basins and $FE_{riv.mi.j}$ and $FE_{riv.ma.j}$ are set at 1 because these sub-basins drain directly to the river mouth (coastal waters). The sub-basins (listed in this table) are named after the main tributaries or a water body that is located close to the outlet. The abbreviations (juT, juC, jmT, jmC, jdT and jdC) are defined according to the modelling approach explained in Supplementary Table 1.

River	Sub-basins and their abbreviation in the model	Area fractions of the main channel (0-1)
Amazon[1]	Purus_juT1_A_jmC_MiddleAmazon	0.12
	Madeira_juT2_A_jmC_MiddleAmazon	0.08
	UpperSubbasin_juT3_A_jmC_MiddleAmazon	0.19
	Negro_juT4_A_jmC_MiddleAmazon	0.09
	Trombetas_juT5_A_jmC_MiddleAmazon	0.00
	MiddleAmazon_jmC_A_jdC_Delta	0.16
	Tapajos_jmT1_A_jdC_Delta	0.14
	Xingu_jmT2_A_jdC_Delta	0.08
Amur	Songhua_juT1_A_jmC_Middlestream	0.21
	Zeya_juT2_A_jmC_Middlestream	0.13
	Argun_juT3_A_jmC_Middlestream	0.20
	MiddlestreamAmur_jmC_A_jdC_DownstreamAmur	0.11
Colorado[2]	Upper_juT1_Achannel_jdC_LowerDelta	0.26
	Gila_juT2_Achannel_jdC_LowerDelta	0.04
Columbia	Columbia_juT1_A_jdC_Delta	0.23
	Snake_juT2_A_jdC_Delta	0.23
Congo	Ubangi_juT1_A_jdC_Delta	0.21
	Songha_juT2_A_jdC_Delta	0.19
	Lualaba_juT3_A_jdC_Delta	0.21
	Kasai_juT4_A_jdC_Delta	0.14
Danube	UpstreamDanube_juT1_A_jmC_MiddlestreamDanube	0.25
	Drava_juT2_A_jmC_MiddlestreamDanube	0.14
	Sava_juT3_A_jmC_MiddlestreamDanube	0.08
	Tisza_juT4_A_jmC_MiddlestreamDanube	0.10
	MiddlestreamDanube_jmC_A_jdC_DownstreamDelta	0.28
	Siret_jmT1_A_jdC_DownstreamDelta	0.06
	Prut_jmT2_A_jdC_DownstreamDelta	0.06
Dnieper	Upstream_juT1_A_jmC_Middlestream	0.19
	Pripet_juT2_A_jmC_Middlestream	0.17
	Desna_juT3_A_jmC_Middlestream	0.13
	Middlestream_jmC_A_jdC_Downstream	0.24
Don	Upstream_juT1_A_jmC_Middstream	0.23
	Khopyor_juT2_A_jmC_Middstream	0.19
	Middstream_jmC_A_jdC_Downstream	0.13
	Donets_jmT_Achannel_jdC_Downstream	0.11
Dvina	Sukhona_juT1_A_jmC_MiddlestreamDvina	0.23
	Vychegda_juT2_A_jmC_MiddlestreamDvina	0.25
	MiddlestreamDvina_jmC_A_jdC_DownstreamDvina	0.10

Supplementary Table 3 continued		
Ganges[3]	UpstreamGanges_juT_Achannel_jmC_MiddlestreamGanges	0.06
	MiddlestreamGanges_jmC_A_jdC_DownstreamGanges	0.12
	Brahmaputra_jmT_A_jdC_DownstreamGanges	0.11
Indus[4]	Sutlej_juT1_A_jmC_MiddlestreamIndus	0.00
	UpstreamIndus_juT2_A_jmC_MiddlestreamIndus	0.16
	MiddlestreamIndus_jmC_A_jdC_DownstreamIndus	0.26
Kolyma[5]	UpstreamKolyma_juT1_A_jmC_MiddlestreamKolyma	0.14
	Omalon_juT2_A_jmC_MiddlestreamKolyma	0.00
	MiddlestreamKolyma_jmC_A_jdC_DownstreamKolyma	0.06
Lena	UpstreamLena_juT1_A_jmC_MiddlestreamLena	0.14
	Aldan_juT2_A_jmC_MiddlestreamLena	0.08
	Vilyuy_juT3_A_jmC_MiddlestreamLena	0.05
	MiddlestreamLena_jmC_A_jdC_DownstreamLena	0.13
Mackenzie	Slave_juT_A_jmC_Hay	0.05
	Hay_jmC_A_jdC_Delta	0.20
	SouthNahanni_jmT1_A_jdC_Delta	0.18
	GreatBearl_jmT2_A_jdC_Delta	0.10
Mekong	MunChi_juT1_A_jmC_MiddlestreamMekong	0.04
	UpstreamMekong_juT2_A_jmC_MiddlestreamMekong	0.26
	MiddlestreamMekong_jmC_A_jdC_DownstreamMekong	0.20
Mississippi	Red_juT1_A_jdC_Lower	0.08
	ArkansasWhite_juT2_A_jdC_Lower	0.13
	Ohio_juT3_A_jdC_Lower	0.18
	Missouri_juT4_A_jdC_Lower	0.20
	Upper_juT5_A_jdC_Lower	0.20
Nelson[6]	Red_juT1_A_jmT_WinnipegLake	0.09
	Saskatchewan_juT2_A_jmT_WinnipegLake	0.08
	WinnipegLake_jmT_A_jdC_Downstream	0.12
Niger	Benue_juT1_Achannel_jdC_Delta	0.05
	SokotoRima_juT2_Achannel_jdC_Delta	0.11
	Middle_juT3_Achannel_jdC_Delta	0.14
	Upper_juT4_Achannel_jdC_Delta	0.17
Nile[7]	BlueNile_juT1_A_jdC_Desert	0.16
	WhiteNile_juT2_A_jdC_Desert	0.16
Ob	UpstreamOb_juT1_A_jdC_DownstreamOb	0.11
	Irtysh_juT2_A_jdC_DownstreamOb	0.07
Parana[8]	Paraguay_juT_A_jmT3_Pilcomayo	0.04
	Pilcomayo_jmT3_A_jdC_Delta	0.15
	Salado_jmT1_A_jdC_Delta	0.07
	Parana_jmT2_A_jdC_Delta	0.15
Pearl[9]	Yujian_juT1_A_jmC_Xijiang	0.10
	Liujiang_juT2_A_jmC_Xijiang	0.06
	Xijiang_jmC_A_jdC1_DeltaZhujiang	0.25
	Beijiang_jmT_A_jdC1_DeltaZhujiang	0.13
Pechora[10]	UpstreamPechora_juT1_A_jdC_DownstreamPechora	0.17
	Usa_juT2_A_jdC_DownstreamPechora	0.17
Supplementary Table 3 continued		

Volga	PermUfa_juT1_A_jmC_MiddlestreamVolga<br>Oka_juT2_A_jmC_MiddlestreamVolga<br>Vyatka_juT3_A_jmC_MiddlestreamVolga<br>UpstreamVolga_juT4_A_jmC_MiddlestreamVolga<br>MiddlestreamVolga_jmC_A_jdC_DownstreamVolga	0.09<br>0.11<br>0.07<br>0.11<br>0.33
Yangtze[11]	Jinsha_juT1_A_juC_UpstreamYangtze<br>Min_juT2_A_juC_UpstreamYangtze<br>Wu_juT3_A_juC_UpstreamYangtze<br>Jialing_juT4_A_juC_UpstreamYangtze<br>UpstreamYangtze_juC_A_jmC_Middlesteam<br>MiddlestreamYangtze_jmC_A_jdC_DeltaYangtze<br>Dongting_jmT1_A_jmC_MiddlestreamYangtze<br>Poyang_jmT2_A_jmC_MiddlestreamYangtze<br>Han_jmT3_A_jmC_MiddlestreamYangtze<br>DownstreamYangtze_jdT_A_jmC_DeltaYangtze	0.32<br>0.30<br>0.19<br>0.21<br>0.33<br>0.42<br>0.25<br>0.00<br>0.17<br>0.10
Yellow[12]	Lanzhou_juT_A_juC_Toudaogual<br>Toudaogual_juC_A_jmC1_Longmen<br>Longmen_jmC1_A_jmC2_Huayuankou<br>Wehe_jmT_A_jmC2_Huayuankou<br>Huanyuankou_jmC2_A_jdC_DeltaYellow	0.39<br>0.15<br>0.24<br>0.27<br>0.93
Yenisey	Selenge_juT1_A_jdC_Downstream<br>Angara_juT2_A_jdC_Downstream<br>PTunguska_juT3_A_jdC_Downstream<br>NTunguska_juT4_A_jdC_Downstream	0.13<br>0.14<br>0.09<br>0.05
Yukon	Tanana_juT1_A_jmC_MiddleYukon<br>UpperYukon_juT2_A_jmC_MiddleYukon<br>UpperYukon_juT3_A_jmC_MiddleYukon<br>MiddleYukon_jmC_A_jdC_DeltaYukon<br>Koyukuk_jmT_A_jdC_DeltaYukon	0.12<br>0.26<br>0.26<br>0.28<br>0.28
Zambezi	Shire_juT1_A_jdC_Delta<br>Kariba_juT2_A_jdC_Delta<br>Kafue_juT3_A_jdC_Delta<br>Luangwa_juT4_A_jdC_Delta	0.05<br>0.21<br>0.21<br>0.17

1: A sub-basin that is classified as an individual river (id=6, name=Araguaia in the model), is not included. The area fraction of a sub-basin of which the outlet is located very close to the main channel (Trombetas_juT5_A_jmC_MiddleAmazon in the table) is assumed to be zero. Pollutants from the Trombetas outlet are exported directly to the main channel of the MiddleAmazon sub-basin because the Trombetas outlet is located very close to the main channel of the MiddleAmazon sub-basin (following the approach of [2]). Therefore the drainage area is assumed to be zero.

2: A sub-basin that is classified as an individual river (id=36, name=Colorado Texas in the model), is not included.

3: Three sub-basins (id=88, 89, 90 in the model) are not included. These sub-basins do not have connections to the surface waters and seas, and thus do not discharge plastics to the seas.

4: Two sub-basins (id=109, 110 in the model) are not included because they do not have connections to the surface waters and seas, and thus do not discharge plastics to seas. The area fraction of a sub-basin of which the outlet is located very close to the main channel (Sutlej_juT1_A_jmC_MiddlestreamIndus in the table) is assumed to be zero. Pollutants from the

Sutlej outlet are exported directly to the main channel of the Middlestream sub-basin because the Sutlej outlet is located very close to the main channel of the Middlestream sub-basin (following the approach of [2]). Therefore the drainage area is assumed to be zero.

5: The area fraction of a sub-basin of which the outlet is located very close to the main channel (Omalon_juT2_A_jmC_MiddlestreamKolyma in the table) is assumed to be zero. Pollutants from the Omalon outlet are exported directly to the main channel of the Middlestream sub-basin because the Omalon outlet is located very close to the main channel of the Middlestream sub-basin (following the approach of [2]). Therefore the drainage area is assumed to be zero.

6: Eight sub-basins (id=171-178 in the model) are specified as "land" and are not included because do not have connections to the surface waters and seas, and thus do not discharge plastics to seas.

7: The drainage area of the Nile River includes four sub-basins. Three of them are included in the table (Blue Nile, White Nile, Desert). The sub-basin "Albarta" (basin id 189 in the model) is not included because it has a Desert, is very dry and does not discharge plastics to sea.

8: A sub-basin is classified as an individual river (id=204, name=Uruguay in the model), and thus is not included in this overview. Another sub-basin with id 207 (in the model) is specified as "land", and thus also is not included because it does not have connections to the surface waters and seas, and thus does not discharge plastics to seas.

9: The drainage area of the Pearl River has six sub-basins. The table present only four sub-basins. This is because these sub-basins drain into downstream areas. The other two sub-basins drain directly to the sea. For example, the table has this sub-basin: Yujian_juT1_A_jmC_Xijiang. It indicates that Yujing (upstream sub-basin) drains into Xijing (middlestream sub-basin). In the other words, plastics are exported by Yujing to Xijiang.

10: The drainage area of the Pechora River includes four sub-basins. Three of them are included here (UpstreamPechora, Usa, DownstreamPechora). The sub-basin "Middlestream" (id=218 in the model) is considered as "land" and does not discharge plastics to sea and thus is not considered in this table.

11: The area fraction of a sub-basin of which the outlet is located very close to the main channel (Poyang_jmT2_A_jmC_MiddlestreamYangtze in the table) is assumed to be zero. Pollutants from the Poyang outlet are exported directly to the main channel of the Delta sub-basin because the Poyang outlet is located very close to the main channel of the Delta sub-basin (following the approach of [2]). Therefore the drainage area is assumed to be zero.

12: jdC for the Yellow River is not included as it has the river mouth. The sub-basin does receive plastics from upstream areas.

Supplementary Table 4. **Sources of the data for model inputs.** The abbreviations are explained in the main text "Methods".

Model inputs	Units	Value	Sources	Code in Supplementary Table 5 for processing methods
For macroplastics				
$P_{MPW.j}$	kg/year	-	Lebreton and Andrady [3]	A
$F_{leakage,j}$	0-1	-	Assigned based on literature	B
$L_{ma.j}$	0-1	0.8	Estimated based on literature and includes retentions along the river banks, fragmentations	C
$Qact_j$ and $Qnat_j$	km^3/year	-	Adjusted to sub-basins using data of [4-6]	D
For microplastics				
FR_f	0-1	0.95	van Wijnen, et al. [7]	-
FR_s	0-1	0.05	van Wijnen, et al. [7]	-
$Area_{land.j}$	km^2	-	Bodirsky, et al. [8]	E
$Area_{average}$	km^2	1264804	Estimated[9]	F
$t_{res.s}$	years	5	van Wijnen, et al. [7]	-
F_{ma}	/year	0.03	van Wijnen, et al. [7]	-
$hw_{mi.j}$	0-1	-	Strokal, et al. [9]	G
$WSdif_{laundry.j}$,<br>$WSdif_{tyres.j}$,<br>$WSdif_{pcp.j}$,<br>$WSdif_{dust.j}$	kg/cap/year	0.12<br>0.18 or 0.018<br>0.0071<br>0.08	Siegfried, et al. [10], Strokal, et al. [9]	H
Urb_{j} and Rur_{j}	People/year	-	Strokal, et al. [9]	-
$fr_{urb.con.j}$,<br>$fr_{rur.con.j}$	0-1	-	Strokal, et al. [9]	-
$L_{mi.j}$	0-1	0.75 or 0.90	Siegfried, et al. [10], Strokal, et al. [9]	I

Supplementary Table 5. **An overview of the methods and their justifications.** These methods are used to process the data for model inputs in Supplementary Table 4.

Code	Processing methods and justifications
A	The global dataset on mismanaged plastic waste (MPW) is taken from Lebreton and Andrady [3] in kg/year on a 30x30 arc seconds resolution for the year 2015. MPW is defined as the combination of inadequately disposed waste (open landfills, dumping), and direct littering.<br><br>We process the MPW data from a 30x30 arc seconds resolution to 10,226 sub-basins as follows. Gridded data are aggregated to sub-basins using the Zonal Statistics to table – Sum tool in ArcMap. This function overlaps two maps (a gridded map with MPW values and a polygon map with delineated sub-basins) and sums MPW values over grids that belong to corresponding sub-basins. The new dataset describes the total MPW entering the terrestrial environment of each sub-basin per year.
B	Not all plastic that enters the environment will reach river systems. MPW can be intercepted by cleaning efforts[11] or can remain in the terrestrial environment by entrapment in soils [12]. A leakage fraction has therefore been added to account for this (equation (3) in Methods of the main text). The leakage fraction is uncertain. A wide range of leakage fractions is used in previously published modelling studies. For example, van Wijnen, et al. [7] assumed a flat rate of 50% of plastic pollution reaching surface waters. Jambeck, et al. [11] and Tramoy, et al. [13] assumed that rates of plastic reaching river systems fall within the 15-40% range, based on a study that examined street cleaning rates in San Francisco, USA[11]. However, Tramoy, et al. [13] noted a large discrepancy between modelled riverine MPW values and observed riverine MPW transport values, which they partially attribute to the assumptions made for the leakage rate.<br><br>We use a range of leakage rates of 1-5%, based on field observations by van Emmerik, et al. [14], Tramoy, et al. [13] and Tramoy, et al. [15] who observed that only small amounts of MPW are exported by rivers to the coastal waters. We assign leakage rates of 1-5% to sub-basins based on the Human Development Index (HDI) of the sub-basins. HDI data was available from[9] (see Supplementary Figure 4.1 in Strokal, et al. [9]. In this study we assume that sub-basins with higher HDI have likely better waste management, leading to less mismanaged plastic waste in the environment and thus in rivers. In general, sub-basins with higher HDI have lower leakage rates compared to sub-basins with lower HDI. Other existing models do this as well, where they use the Gross Domestic Products of a basin to assign a leakage rate[3]. We use HDI since this is a wider definition of human development, which combines Gross Domestic Product, life expectancy, and education [16].

Supplementary Table 5 continued

B	We assign the leakage rates of 1-5% based on HDI as follows:<br>○ First, we make categories of HDI based on the HDI classification used in the 2009 United Nations Human Development Report (UNHDR)[17]. These categories are low (0-0.499), medium (0.500-0.799), high (0.800-0.899) and very high (0.900-1) human development.<br>○ Second, we assign sub-basins to those categories. In this way, we identify sub-basins with low, medium, high and very high human developments. It is assumed that sub-basins with a low HDI have a high fraction of MPW reaching rivers and vice versa. HDI values for sub-basins are in Supplementary Figure 3.<br>○ A full overview of the HDI and the associated leakage rates:<br>HDI range / HDI Classification / Leakage rate:<br>0-0.499 / Low / 5%<br>0.500 – 0.799 / Medium / 4%<br>0.800 – 0.899 / High / 2%<br>0.900 – 1 / Very High / 1%
C	The processes that affect the sedimentation of macroplastics are different compared to microplastics. Schöneich-Argent, et al. [18] is one of the first studies that quantified plastic transport and accumulation in all river compartments (e.g. floating, suspended in the water column, in biota, sediment, and deposited on riverbanks) as identified by [19-21]. Schöneich-Argent, et al. [18] did this for three rivers in Germany: the Ems, Weser, and Elbe rivers (Supplementary Table 6). To estimate the fraction of macroplastics that are removed from riverine transport due to sedimentation ($L_{ma.j}$), we compare the mass of plastic being transported (e.g. floating plastic, plastic in the water column, riverbed transport) to the accumulation rate of plastics on riverbanks (Supplementary Table 6). The mean ratio of transport versus retained for all three rivers is calculated. This is done by first dividing the plastic that accumulates on riverbanks by total plastics for the river (mean total transport and mean daily accumulation on riverbanks) to determine the fraction of plastic that is retained for each river. We then calculate the mean for all rivers. This results in a rough estimate for macroplastic retentions in rivers at 0.8 (fraction between 0 and 1). We use 0.8 in our model for sub-basins.
D	Natural river discharges ($Qnat_j$) were derived for the 0.5˚ grid that is located at the outlet of the sub-basin. The data is from the VIC (Variable Infiltration Capacity) hydrological model for the historical period up to 2010 (see Supplementary Table 4). We calculated river discharges for 2010 by averaging the data over the 30 years of the period 1980-2010. Then, we calculated the sub-basin-specific water discharges by subtracting the upstream discharges if needed. Actual river discharges ($Qact_j$) for 2010 are calculated using the ratio of Qnat and Qact from Mayorga, et al. [22] and natural river discharges from the VIC hydrological model (see above).

HDI range	HDI Classification	Leakage rate
0-0.499	Low	5%
0.500 – 0.799	Medium	4%
0.800 – 0.899	High	2%
0.900 – 1	Very High	1%

Supplementary Table 5 continued	
E	Data were available for the word at the grid cell resolution of 0.5˚. This data was aggregated to sub-basins using ArcGIS. This was done by summing the land area over grids of the corresponding sub-basins.
F	The 50 rivers with the largest drainage areas were taken to average their areas.
G	The treatment fractions were directly taken from Strokal, et al. [9] who processed the country data for primary, secondary and tertiary treatment and their removal efficiencies to sub-basins. For this, Strokal, et al. [9] used the approach of Siegfried, et al. [10] to determine the average removal of microplastics during treatment. Details can be found in the supporting information of Strokal, et al. [9].
H	$WSdif_{tyres.j}$ depends on HDI following Strokal, et al. [9]<br>○ If HDI>0.785, $WSdif_{tyres.j}$ = 0.18<br>○ If HDI<0.785, then $WSdif_{tyres.j}$ = 0.018<br>An assumption is that sub-basins with higher HDI most likely are more developed and can afford cars. In those sub-basins, the production of microplastics from car tyre wear is assumed to be higher. HDI data for sub-basins are in Supplementary Figure 3. Siegfried, et al. [10] were the first to calculate river exports of microplastics. They used one single parameter to represent the car tyre rates. Strokal, et al. [9] took that approach and improved it by adding some spatial variability among the sub-basins. This was done based on HDI assuming that some sub-basins are richer than others and can afford more cars (implying more microplastic production) than others.
I	According to the approach of Siegfried, et al. [10]:<br>○ If the sub-basin covers ≥ 4 grids, then $L_{mi.j}$ = 0.90<br>○ If the sub-basin covers < 4 grids, then $L_{mi.j}$ = 0.75<br>An assumption is that larger sub-basins likely retain more plastics compared to smaller sub-basins that cover less than 4 grids of 0.5˚ (approximately 10,000 km^2).

Supplementary Table 6. **Macroplastic export to coastal waters and accumulation rates in items per day (n/day) in three German rivers (Ems, Weser, Elbe), and the retention factor.** An estimate for the retention factor is based on the ratio of exported macroplastic to coastal waters versus accumulated macroplastics in the rivers, adapted from Schöneich-Argent, et al. [18]. The mean surface, column, and riverbed transport values are the average fluxes found by Schöneich-Argent, et al. [18] of macroplastic pollution in each compartment in items per day over six months. The mean total transport is the transport of macroplastic in the surface, column and riverbed compartments combined. The mean accumulation on riverbanks is the observed values of accumulation of plastics on riverbanks in items per day over six months as observed by Schöneich-Argent, et al. [18], extrapolated for the full river length of the river. The ratio deposited is the ratio between the mean total transport and the mean daily accumulation.

	Mean surface transport (n/day)	Mean column transport (n/day)	Mean riverbed transport (n/day)	Mean total transport (n/day)	Mean daily accumulation riverbanks (n/day)	Ratio retained (0-1)
Ems	263	203	2	468	33390	0.9
Weser	1309	212	3	1524	20736	0.9
Elbe	98309	1487	8	99803	299200	0.7
Mean						0.8*

Supplementary Table 7. **Observed river export of macroplastics in the 15 existing studies (10^3 ton/year).** Where necessary, values reported were linearly extrapolated to determine annual exports. Locations of the rivers are shown in Supplementary Figure 4.

River name	Study	Macroplastics (10^3 ton/year)	Code in Supplementary Figure 4
Ems	Schöneich-Argent, et al. [18]	0.9	A
Weser	Schöneich-Argent, et al. [18]	1.3	B
Elbe	Schöneich-Argent, et al. [18]	15	C
Rhine	Vriend, et al. [23]	0.5	D
Rhine	van der Wal, et al. [24]	6	D
Rhone	Castro-Jiménez, et al. [25]	0.7	E
Saigon	van Emmerik, et al. [14]	1100	F
Jakarta area	Van Emmerik, et al. [26]	2100	G
Seine	Tramoy, et al. [13]	1100	H
Danube	Lechner, et al. [27]	1533	I
Danube	van der Wal, et al. [24]	530	I
Danube	Gonzalez-Fernandez [28]	3	I
Po	van der Wal, et al. [24]	120	J
llobregat + Besos	Schirinzi, et al. [29]	0.4	M
Pahang	van Calcar and van Emmerik [30]	38	N
Chao praya	van Calcar and van Emmerik [30]	118	O
Klang	van Calcar and van Emmerik [30]	165	P
Dniester	Gonzalez-Fernandez [28]	2	Q
Don	Gonzalez-Fernandez [28]	0.4	R
Chorokni	Gonzalez-Fernandez [28]	1	S
Natanebi + Rioni + Supsa	Gonzalez-Fernandez [28]	3.3	T
Taslidere	Gonzalez-Fernandez [28]	1.6	U
Firtina	Gonzalez-Fernandez [28]	1.9	V
Kuantan	van Calcar and van Emmerik [30]	756	W
Jones fall	Lindquist [31]	252	X
Meycuayan + Tullahan + Pasig	Meijer, et al. [32]	13812	Y
Tiber	Crosti, et al. [33]	1721	Z
Motagua	Meijer, et al. [32]	155	AA

Supplementary Table 8. **Comparison of the MARINA-Plastics model outputs for annual global macroplastic exports with four macroplastic export models (million tonnes/year).** Lebreton refers to the model developed by Lebreton, et al. [34], Jambeck refers to the model developed by Jambeck, et al. [11], Schmidt refers to the model developed by Schmidt, et al. [35], Meijer refers to the model developed by Meijer, et al. [32], Mai (1) refers to the model developed by Mai, et al. [36], Mai (2) refers to the model of Mai, et al. [37], Borelle refers to the model developed by Borrelle, et al. [38], Lau refers to the model developed by Lau, et al. [39] and Zhang refers to Zhang, et al. [40]. Nakayama refer to Nakayama and Osako [41]. The description of the MARINA-Plastics model of this study is provided in the "Methods" of the main manuscript.

Model	Value (million ton/year)
MARINA-Plastics (this study)	0.5
Lebreton	1.1
Jambeck	9.6
Schmidt	0.4
Meijer	0.8
Mai (1)	0.1
Borrelle	19.3
Lau	22
Nakayama	0.3-1.5*
Mai (2)	0.15-0.53
Zhang	0.70 (0.13 to 3.8 as a 95% confidence interval)

*The range is for three cases for macroplastics reflecting three different densities: 1001.0 kg/m^3 (case-a), 1000.001 kg/m^3 (case-b), and 1000.0001 kg/m^3 (case-c).

Supplementary Table 9. **Comparison of the MARINA-Plastics model in this study with other (selected) modelling approaches.** Point sources are, for example, sewage systems that are the primary sources of microplastics in rivers. Diffuse sources are, for example, mismanaged waste that contributes macroplastics in rivers or microplastics in rivers from the degradation of macroplastics (this is the secondary source of microplastics in rivers).

Model	Modelling approach	Level of detail	Applied	Calculate the source attribution	
				Point sources	Diffuse sources
MARINA-Plastics (this study)	Process-based	2010-2015, macro, micro, sub-basins	Globally (10,226 sub-basins)	Yes	Yes
Jambeck, et al. [11]	Empirically-based	2010, macro, coasts	Globally	No	Yes
Lebreton, et al. [34]	Empirically- based	2017, macro, micro, basins	Globally	No	Yes
Meijer, et al. [32]	Probability-based	2015, macro, basins	Globally	No	Yes
Schmidt, et al. [35]	Mismanaged waste as a predictor for plastic flux	2010, macro, basins	Globally	No	Yes
Turrell [42]	Marine environment centred	2008-2017, macro, micro, Scottish coasts	Regionally	No	Yes
Tramoy, et al. [13]	Empirically-based	2018-2019, macro, Seine estuary	Regionally	No	Yes
Jang, et al. [43]	Stock and flow	2012, macro, South Korea	Regionally	No	Yes
Nihei, et al. [44]	Macro based on microplastic presence	2018-2019, macro, micro, Japan	Regionally	No	Yes
Mai, et al. [36]	Empirically-and correlation-based	2010-2050, macro	Globally	No	Yes*
Nakayama and Osako [41]**	Probability-based	2015, macro, micro	325 major rivers	Yes	Yes
Mai, et al. [37]	R_2O model based on population, humna development index, waste generation	2016, macro, countries	161 countries	No	Yes
Zhang, et al. [40]	A threedimensional Euler-based global ocean plastic model based on surface ocean plastic abundance data	1950 to 2018, macro, micro, a resolution of 2° × 2.5° horizontally with 22 vertical levels, and a time step of 4 h.	Global	Yes**	Yes**

*Mismanaged solid waste with a focus of 1518 rivers in the world; **the authors simulated the macro- and microplastic fluxes to rivers and oceans based on three cases. For macroplastics, the three cases reflected three different densities: 1001.0 kg/m^3 (case-a), 1000.001 kg/m^3 (case-b), and 1000.0001 kg/m^3 (case-c). For microplastics, the three cases reflected per capita emissions: the same value for the whole world (case-a, [10]), diferent value for the whole world (case-b, [45]) and the different values for each region (case-c, [7]). **No explicit source attribution in riverine plastic export.

Supplementary Table 10. **A set up of the sensitivity analysis for the MARINA-Plastics model**. It was inspired by Strokal, et al. [9]. The description of the abbreviations and the equations can be found in "Methods" in the main text. Macro and micro refer to macroplastics and microplastics, respectively.

Number	Model inputs	Used in equations	Changed relative to the original run	Affects inputs to rivers		Affects river export fractions	
				Macro	Micro	Macro	Micro
Point-source inputs to rivers							
1	$fr_{\mathrm{urb.con.j}}$	(14)	+10%				
2	$WSdif_{tyres.j}$	(12)	+10%				
3	$WSdif_{pcp.j}$	(12)	+10%				
4	$WSdif_{dust.j}$	(12)	+10%				
5	$WSdif_{laundry.j}$	(12)	+10%				
6	$hw_{mi.j}$	(11)	+10%				
Diffuse-source inputs to rivers							
7	$Area_{average}$	(10a), (10b)	+10%				
8	$P_{MPW.j}$	(3)	+10%				
9	$F_{leakage,j}$	(3)	+10%				
10	F_{ma}	(7)	+10%				
11	$t_{res.f.j}$	(7)	+10%				
12	$t_{res.s}$	(7)	+10%				
River export fractions (reflect retentions in rivers)							
13	$FE_{riv.mi.o.j}$	(6)	+10%				
14	$FE_{riv.mi.m.j}$	(6)	+10%				
15	$FE_{riv.ma.o.j}$	(2)	+10%				
16	$FE_{riv.ma.m.j}$	(2)	+10%				

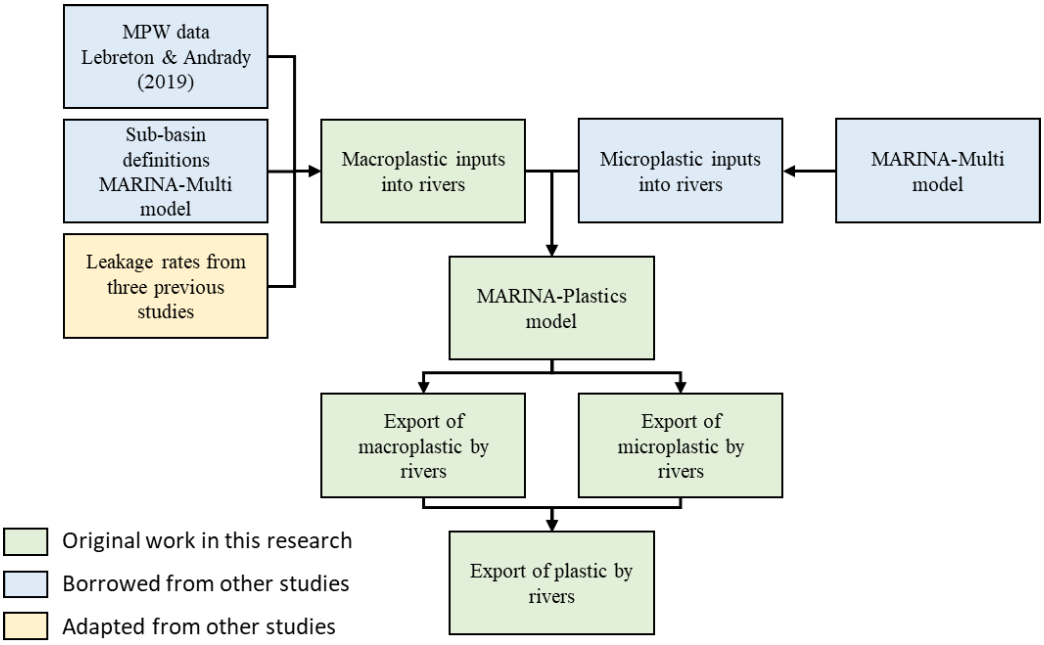

Supplementary Figure 1. **Schematic overview of the inputs and outputs of the MARINA-Plastics model.** Green boxes represent elements that are created specifically for the MARINA-Plastics model, and blue boxes represent elements that have been borrowed from other studies. Yellow boxes represent elements that have been adapted from other studies. The MARINA-Multi model (version 1.0) refers to the model presented by Strokal, et al. [9]. MARINA is short for a Model to Assess River Inputs of pollutaNts to seAs.

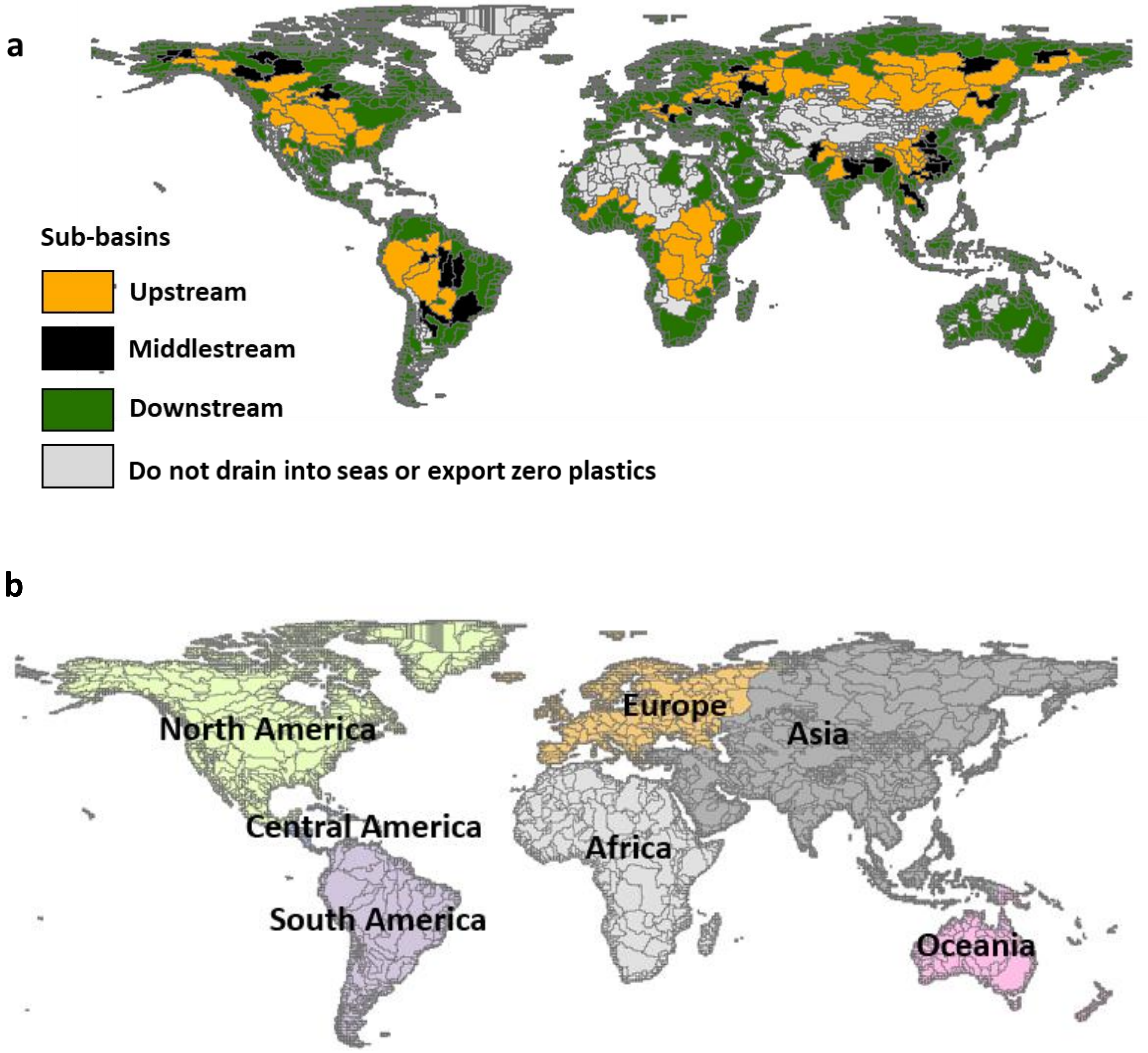

Supplementary Figure 2. **Sub-Basins of the MARINA-Plastics model. a** Locations of up-, middle- and downstream sub-basins.**b** their continents. These sub-basins are defined in an earlier study[9] based on the water flow direction and land mask from a hydrological Variable Infiltration Capacity model [4-6] and on the hydrological characteristics of the basins. The study area has 10,226 sub-basins. Of these, riverine plastics from 6,620 sub-basins reach coastal seas (indicated in different colours in Supplementary Fig.2a). The other sub-basins (indicated in grey in Supplementary Fig. 2a) either do not drain into coastal seas (1,318 sub-basins) or export zero plastics (2,288 sub-basins).

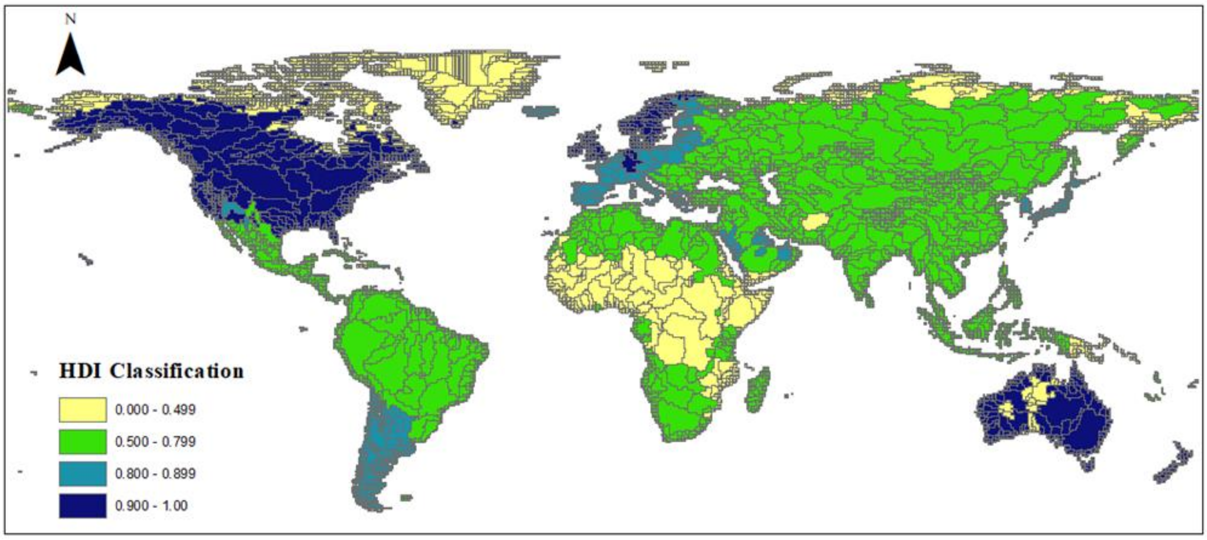

Supplementary Figure 3. **Human Development Index for sub-basins.** The data are from Strokal, et al. [9]. The classification is according to the 2009 United Nations Human Development Report (UNHDR)[17].

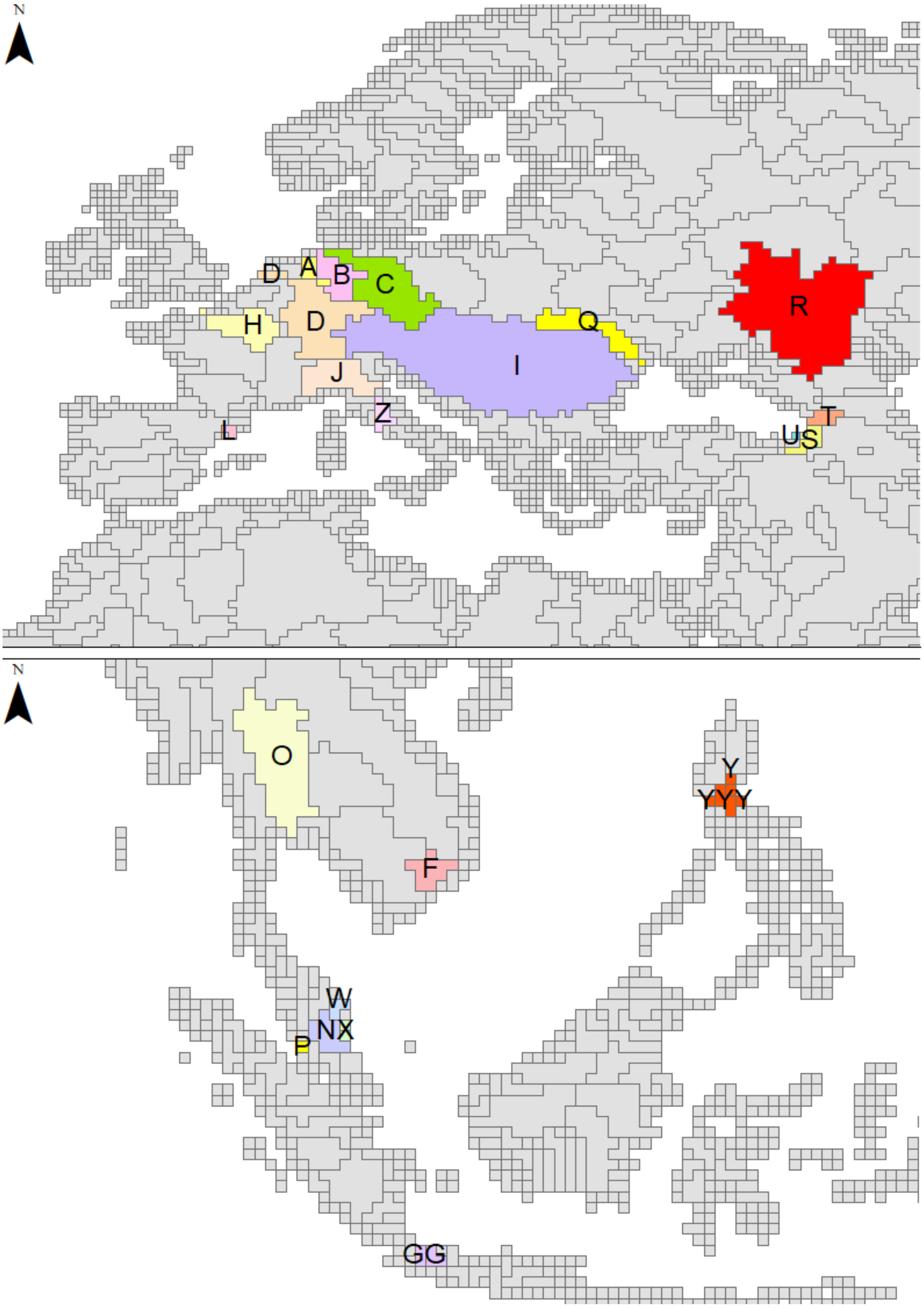

Supplementary Figure 4. **Locations of observational studies were used to evaluate the MARINA-Plastics model.** The letter codes refer to studies or rivers in Supplementary Table 7.

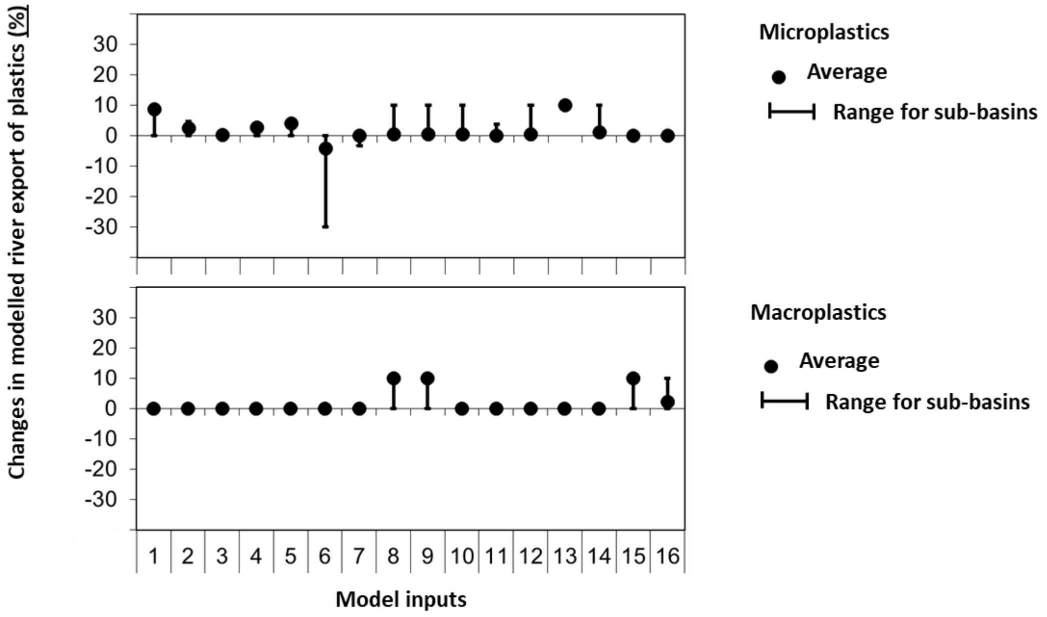

Supplementary Figure 5. **Results of the sensitivity analysis for the MARINA-Plastics model.** The results are shown as the percentage change in river export of macro- and microplastics relative to the original model run. The sensitivity analysis setup and the description of 16 model inputs are provided in Supplementary Table 10. The graphs exclude sub-basins with no data or with zero values (in that population and waste managed are zero).

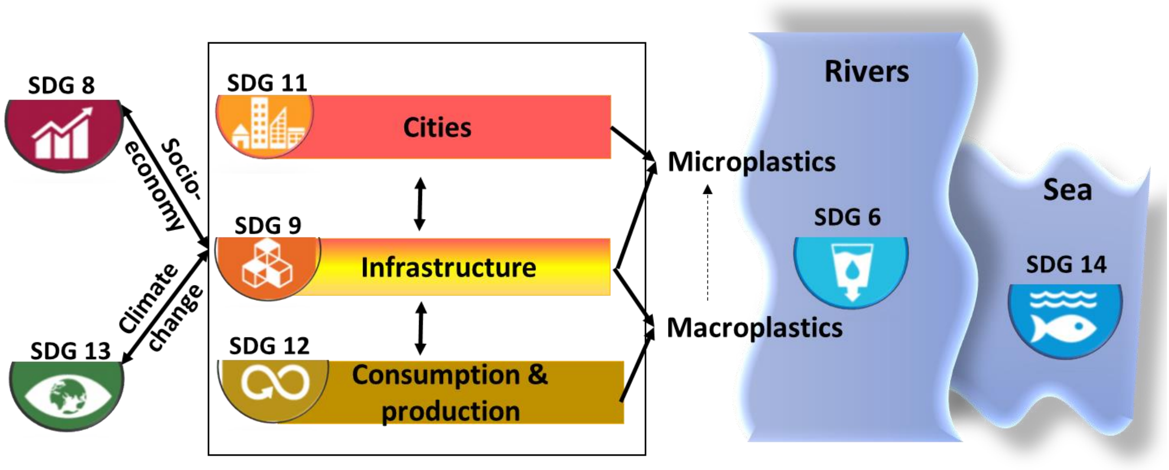

Supplementary Figure 6. **Interactions in pollution control for plastics between sustainable development goals.** a focus IS on dominant sources of macro- and microplastics from the findings of our study.

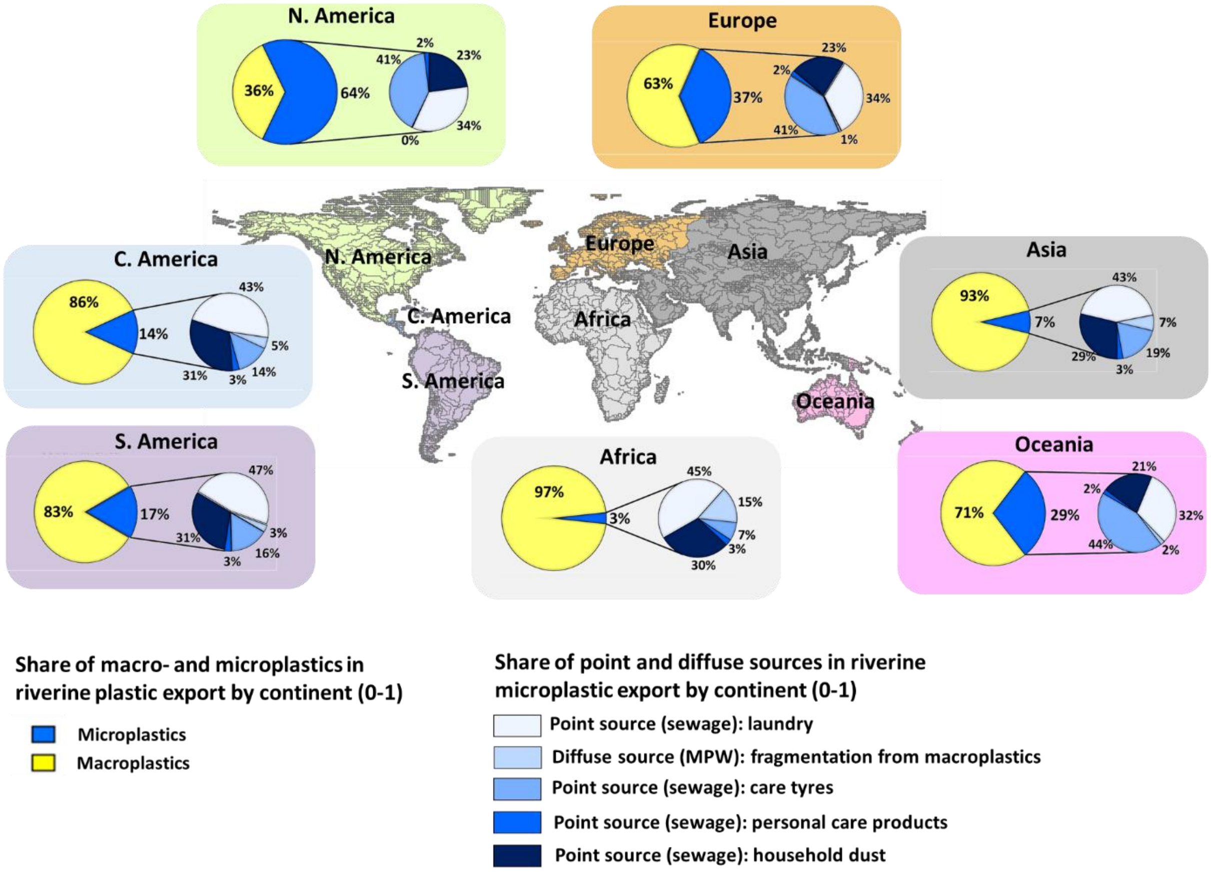

Supplementary Figure 7. **Sources of riverine plastic exports by continent (shares, %s).** N, S and C are short for North, Central and South, respectively. Source: the MARINA-Plastics model (see "Methods" in the main manuscript).

Supplementary Disucssion

Some uncertainties are associated with large-scale data and processing this data to sub-basins (see Supplementary Table 5). We used existing datasets[3-5,9] that are often not at the sub-basin scale. Examples are the country-specific rates of the population connected to sewage systems. For microplastics, data was processed to sub-basins by Strokal, et al. [9] using the population density at the grid of 0.5˚ for model inputs that are country- or region-specific (e.g. national connection rates to sewage). Population density is a widely used method to downscale data for large-scale water quality models[46,47]. However, this method does not explicitly account for technological developments and treatments. For macroplastics, mismanaged plastic waste from Lebreton and Andrady [3] was aggregated into sub-basins. Retentions of plastics in rivers were calculated using 30-year averaged river discharges from a hydrological model and associated model inputs (see details in "Methods"). Large-scale data has uncertainties, contributing to uncertainties in plastic pollution levels. The same holds not only for plastics but also for other pollutants that are modelled by large-scale models[46,48]. We used existing data that is available to us to model river exports of plastics.

Weiss, et al. [49] discussed the uncertainties associated with the conversion of microplastic numbers to mass, different sampling techniques and the relationship between microplastics and mismanaged plastic waste that is often used to estimate microplastic fluxes in seas. They show that the microplastic fluxes can vary by several orders of magnitude depending on conversion factors (from observed numbers of microplastics in seas to their mass) and type of sampling techniques. This challenges the validation of large-scale models such as MARINA-Plastics. Furthermore, the relationship between microplastics and mismanaged plastic waste is uncertain [49], but used in existing large-scale estimates of plastics in seas[35]. In our study, river exports of microplastics are calculated from diffuse and point sources. Diffuse sources reply to the data for mismanaged plastic waste.

Other limitations are associated with the seasonality and sources. Floods and extreme events can influence plastic export[50], which is not part of our study. We account for the sources of plastics in water associated with point source inputs (car tyres, laundry, PCP, household dust) and diffuse-source inputs, but ignore plastics in rivers from agricultural films[12], industries[51], ships[52] or deposited from the air[53]. Thus, our input of plastics from rivers to seas might be underestimated. Nevertheless, we believe that we account for the most relevant sources of macro- and microplastics associated with urbanization and waste management, which is in line with other studies[3,39,42,54].

To better understand the impact of uncertainties, we performed a sensitivity analysis (Supplementary Table 10 and Supplementary Figure 5). We selected 16 model inputs reflecting the calculations of the point- and diffuse sources of plastics to rivers and their river exports (see the list in "Methods" and Supplementary Table 10). We altered those inputs by +-10%. Our results indicate a low sensitivity of the amount of plastics exported to seas to changes in most inputs (Supplementary Figure 5). River export of microplastics from sub-basins is somewhat sensitive (0-30%) to changes in treatment removals (model input 6 in Supplementary Figure 5). For river export of macroplastics, this holds for changes in mismanaged solid waste and leakage rates (model inputs 8 and 9 in Supplementary Figure 5). A similar conclusion is for retentions of macro- and microplastics in rivers (model inputs 13 and 15 in Supplementary Figure 5). This implies that the model outputs are somewhat sensitive to removals during wastewater treatment, mismanaged waste, leakage rates and river retentions. Some of these model inputs are based on expert knowledge, limited literature and human development index (e.g. leakage rates, see Supplementary Table 5). This contributes to uncertainties in model outputs. Nevertheless, we believe that uncertainties in model inputs and modelling approaches do not largely affect our messages on a global and regional scale. However, for local analysis of plastic pollution (e.g. national scale or smaller), the model needs to be further validated and checked for specific local conditions.

Supplementary Note

Class I sub-basins are largely polluted with sewage wastewater discharging microplastics to rivers. These sub-basins have many urban areas with high rates of sewage connections. As a result, most wastewater from streets (microplastics from car tires) and houses (microplastics from laundry, personal care products, dust) enters sewage systems and then treatment facilities. Depending on treatment levels, microplastics are released into rivers. These are mainly well-developed sub-basins with good collection systems of solid waste. Thus, mismanaged waste production is relatively low, and contributes less to macroplastics in rivers. These sub-basins are largely located in Europe, North America, and Oceania.

Class II sub-basins have more mismanaged solid waste than Class I. This solid waste contributes considerably to macroplastics in rivers. These sub-basins are not as developed as Class I sub-basins and have relatively poor solid waste management. These sub-basins have also a lower connection to sewage systems compared to Class I sub-basins. As a result, more macroplastics (as mass) enter rivers from mismanaged solid waste and less microplastics (as mass) from sewage systems. Thus, macroplastics (as mass) dominate in Class II sub-basins. These sub-basins are largely located in Asia, South America, and Africa.

Class III sub-basins have high sewage connection rates and a relatively high production of mismanaged solid waste. High sewage connection rates mean that wastewater from streets and houses is collected and treated (depending on treatment levels). The plastics in this wastewater are mainly microplastics. A high production of mismanaged solid waste means that waste management is poor and considerable amounts of macroplastics can enter rivers. As a result, these sub-basins are characterized by the dominant amounts of both macro and microplastics in their rivers. These sub-basins are largely located in some parts of Europe, North and South America (e.g., along the coast, see Figure 2 in the manuscript).

Supplementary References

1 Wang, M., Kroeze, C., Strokal, M., van Vliet, M. T. & Ma, L. Global change can make coastal eutrophication control in China more difficult. *Earth's Future* **8**, 1-19, (2020).

2 Strokal, M., Kroeze, C., Wang, M., Bai, Z. & Ma, L. The MARINA model (Model to Assess River Inputs of Nutrients to seAs): model description and results for China. *Science of the Total Environment* **562**, 869-888, (2016).

3 Lebreton, L. & Andrady, A. Future scenarios of global plastic waste generation and disposal. *Palgrave Communications* **5**, 1-11, (2019).

4 van Vliet, M. T., Franssen, W. H., Yearsley, J. R., Ludwig, F., Haddeland, I., Lettenmaier, D. P. & Kabat, P. Global river discharge and water temperature under climate change. *Global Environmental Change* **23**, 450-464, (2013).

5 Van Vliet, M., Ludwig, F., Zwolsman, J., Weedon, G. & Kabat, P. Global river temperatures and sensitivity to atmospheric warming and changes in river flow. *Water Resources Research* **47**, W02544, (2011).

6 Fekete, B. M., Wisser, D., Kroeze, C., Mayorga, E., Bouwman, L., Wollheim, W. M. & Vörösmarty, C. Millennium ecosystem assessment scenario drivers (1970–2050): climate and hydrological alterations. *Global Biogeochemical Cycles* **24**, GB0A12, (2010).

7 van Wijnen, J., Ragas, A. M. J. & Kroeze, C. Modelling global river export of microplastics to the marine environment: Sources and future trends. *Science of The Total Environment* **673**, 392-401, (2019).

8 Bodirsky, B. L., Popp, A., Weindl, I., Dietrich, J. P., Rolinski, S., Scheiffele, L., Schmitz, C. & Lotze-Campen, H. N₂O emissions from the global agricultural nitrogen cycle – current state and future scenarios. *Biogeosciences* **9**, 4169-4197, (2012).

9 Strokal, M., Bai, Z., Franssen, W., Nynke, H., Koelmans, A. A., Ludwig, F., Ma, L., van Puijenbroek, P., Spanier, J. E., Vermeulen, L. C., van Vliet, M. T. H., van Wijnen, J. & Kroeze, C. Urbanization: an increasing source of multiple pollutants to rivers in the 21st century. *Urban Sustainability* **1**, 24, (2021).

10 Siegfried, M., Koelmans, A. A., Besseling, E. & Kroeze, C. Export of microplastics from land to sea. A modelling approach. *Water Research* **127**, 249-257, (2017).

11 Jambeck, J. R., Geyer, R., Wilcox, C., Siegler, T. R., Perryman, M., Andrady, A., Narayan, R. & Law, K. L. Plastic waste inputs from land into the ocean. *Science* **347**, 768-771, (2015).

12 Piehl, S., Leibner, A., Löder, M. G., Dris, R., Bogner, C. & Laforsch, C. Identification and quantification of macro-and microplastics on an agricultural farmland. *Scientific reports* **8**, 1-9, (2018).

13 Tramoy, R., Gasperi, J., Dris, R., Colasse, L., Fisson, C., Sananes, S., Rocher, V. & Tassin, B. Assessment of the plastic inputs from the seine basin to the sea using statistical and field approaches. *Frontiers in Marine Science* **6**, 151, (2019).

14 van Emmerik, T., Strady, E., Kieu-Le, T.-C., Nguyen, L. & Gratiot, N. Seasonality of riverine macroplastic transport. *Scientific reports* **9**, 1-9, (2019).

15 Tramoy, R., Gasperi, J., Colasse, L., Silvestre, M., Dubois, P., Noûs, C. & Tassin, B. Transfer dynamics of macroplastics in estuaries–new insights from the Seine estuary: part 2. Short-term dynamics based on GPS-trackers. *Marine Pollution Bulletin* **160**, 111566, (2020).

16 Bleys, B. Beyond GDP: Classifying alternative measures for progress. *Social Indicators Research* **109**, 355-376, (2012).

17 Klugman, J. Human development report 2009. Overcoming barriers: Human mobility and development. *Overcoming Barriers: Human Mobility and Development (October 5, 2009). UNDP-HDRO Human Development Reports*, (2009).

18 Schöneich-Argent, R. I., Dau, K. & Freund, H. Wasting the North Sea?–A field-based assessment of anthropogenic macrolitter loads and emission rates of three German tributaries. *Environmental Pollution*, 114367, (2020).

19 van Emmerik, T., Vriend, P. & Roebroek, J. An evaluation of the River-OSPAR method for quantifying macrolitter on Dutch riverbanks (Report). *Wagemningen, Wageningen University, The Netherlands.* *https://doi.org/10.18174/519776*, 86 pp., (2020).
20 van Emmerik, T., Van Klaveren, J., Meijer, L. J. J., Krooshof, J. W., Palmos, D. A. A. & Tanchuling, M. A. Manila river mouths act as temporary sinks for macroplastic pollution. *Frontiers in Marine Science* **7**, 770, (2020).
21 van Emmerik, T. & Schwarz, A. Plastic debris in rivers. *Wiley Interdisciplinary Reviews: Water* **7**, e1398, (2020).
22 Mayorga, E., Seitzinger, S. P., Harrison, J. A., Dumont, E., Beusen, A. H. W., Bouwman, A. F., Fekete, B. M., Kroeze, C. & Van Drecht, G. Global Nutrient Export from WaterSheds 2 (NEWS 2): Model development and implementation. *Environmental Modelling & Software* **25**, 837-853, (2010).
23 Vriend, P., Van Calcar, C., Kooi, M., Landman, H., Pikaar, R. & Van Emmerik, T. Rapid assessment of floating macroplastic transport in the Rhine. *Frontiers in Marine Science* **7**, 10, (2020).
24 van der Wal, M., van der Meulen, M., Tweehuijsen, G., Peterlin, M., Palatinus, A. & Kovac Viršek, M. SFRA0025: Identification and assessment of riverine input of (Marine) litter. *Report for Michail Papadoyannakis, DG Environment, United Kingdom*, 186, (2015).
25 Castro-Jiménez, J., González-Fernández, D., Fornier, M., Schmidt, N. & Sempéré, R. Macro-litter in surface waters from the Rhone River: Plastic pollution and loading to the NW Mediterranean Sea. *Marine pollution bulletin* **146**, 60-66, (2019).
26 Van Emmerik, T., Loozen, M., Van Oeveren, K., Buschman, F. & Prinsen, G. Riverine plastic emission from Jakarta into the ocean. *Environmental Research Letters* **14**, 084033, (2019).
27 Lechner, A., Keckeis, H., Lumesberger-Loisl, F., Zens, B., Krusch, R., Tritthart, M., Glas, M. & Schludermann, E. The Danube so colourful: A potpourri of plastic litter outnumbers fish larvae in Europe's second largest river. *Environmental Pollution* **188**, 177-181, (2014).
28 Gonzalez-Fernandez. Anthropogenic litter input through rivers in the black sea. . *Marine litter in the black sea, Turkish Marine research foundation (TUDAV). Publication no:56, Istanbul, Turkey*, (2020).
29 Schirinzi, G. F., Köck-Schulmeyer, M., Cabrera, M., González-Fernández, D., Hanke, G., Farré, M. & Barceló, D. Riverine anthropogenic litter load to the Mediterranean Sea near the metropolitan area of Barcelona, Spain. *Science of The Total Environment* **714**, 136807, (2020).
30 van Calcar, C. J. v. & van Emmerik, T. H. M. v. Abundance of plastic debris across European and Asian rivers. *Environmental Research Letters* **14**, 124051, (2019).
31 Lindquist, A. Baltimore’s Mr. Trash wheel. *J. Ocean Technol* **11**, 28-35, (2016).
32 Meijer, L. J., van Emmerik, T., van der Ent, R., Schmidt, C. & Lebreton, L. More than 1000 rivers account for 80% of global riverine plastic emissions into the ocean. *Science Advances* **7**, eaaz5803, (2021).
33 Crosti, R., Arcangeli, A., Campana, I., Paraboschi, M. & González-Fernández, D. ‘Down to the river’: amount, composition, and economic sector of litter entering the marine compartment, through the Tiber river in the Western Mediterranean Sea. *Rendiconti Lincei. Scienze Fisiche e Naturali* **29**, 859-866, (2018).
34 Lebreton, L. C., Van der Zwet, J., Damsteeg, J.-W., Slat, B., Andrady, A. & Reisser, J. River plastic emissions to the world's oceans. *Nature Communications* **8**, 15611, (2017).
35 Schmidt, C., Krauth, T. & Wagner, S. Export of Plastic Debris by Rivers into the Sea. *Environmental Science & Technology* **51** 12246–12253, (2017).
36 Mai, L., Sun, X.-F., Xia, L.-L., Bao, L.-J., Liu, L.-Y. & Zeng, E. Y. Global riverine plastic outflows. *Environmental Science & Technology* **54**, 10049-10056, (2020).
37 Mai, L., Sun, X. & Zeng, E. Y. Country-specific riverine contributions to marine plastic pollution. *Science of The Total Environment* **874**, 162552, (2023).

38 Borrelle, S. B., Ringma, J., Law, K. L., Monnahan, C. C., Lebreton, L., McGivern, A., Murphy, E., Jambeck, J., Leonard, G. H. & Hilleary, M. A. Predicted growth in plastic waste exceeds efforts to mitigate plastic pollution. *Science* **369**, 1515-1518, (2020).

39 Lau, W. W., Shiran, Y., Bailey, R. M., Cook, E., Stuchtey, M. R., Koskella, J., Velis, C. A., Godfrey, L., Boucher, J. & Murphy, M. B. Evaluating scenarios toward zero plastic pollution. *Science* **369**, 1455-1461, (2020).

40 Zhang, Y., Wu, P., Xu, R., Wang, X., Lei, L., Schartup, A. T., Peng, Y., Pang, Q., Wang, X. & Mai, L. Plastic waste discharge to the global ocean constrained by seawater observations. *Nature Communications* **14**, 1372, (2023).

41 Nakayama, T. & Osako, M. The flux and fate of plastic in the world's major rivers: Modelling spatial and temporal variability. *Global and Planetary Change*, 104037, (2023).

42 Turrell, W. R. Estimating a regional budget of marine plastic litter in order to advise on marine management measures. *Marine pollution bulletin* **150**, 110725, (2020).

43 Jang, Y. C., Lee, J., Hong, S., Mok, J. Y., Kim, K. S., Lee, Y. J., Choi, H.-W., Kang, H. & Lee, S. Estimation of the annual flow and stock of marine debris in South Korea for management purposes. *Marine pollution bulletin* **86**, 505-511, (2014).

44 Nihei, Y., Yoshida, T., Kataoka, T. & Ogata, R. High-Resolution Mapping of Japanese Microplastic and Macroplastic Emissions from the Land into the Sea. *Water* **12**, 951, (2020).

45 Boucher, J., Billard, G., Simeone, E. & Sousa, J. The marine plastic footprint. *n: Global Marine and Polar Programme. IUCN, Switzerland* https://doi.org/10.2305/IUCN.CH.2020.01.en 69 pp., (2020).

46 Beusen, A., Van Beek, L., Bouwman, L., Mogollón, J. & Middelburg, J. Coupling global models for hydrology and nutrient loading to simulate nitrogen and phosphorus retention in surface water–description of IMAGE–GNM and analysis of performance. *Geoscientific Model Development* **8**, 4045-4067, (2015).

47 van Puijenbroek, P. J. T. M., Beusen, A. H. W. & Bouwman, A. F. Global nitrogen and phosphorus in urban waste water based on the Shared Socio-economic pathways. *Journal of Environmental Management* **231**, 446-456, (2019).

48 Vermeulen, L. C., van Hengel, M., Kroeze, C., Medema, G., Spanier, J. E., van Vliet, M. T. & Hofstra, N. Cryptosporidium concentrations in rivers worldwide. *Water research* **149**, 202-214, (2019).

49 Weiss, L., Ludwig, W., Heussner, S., Canals, M., Ghiglione, J.-F., Estournel, C., Constant, M. & Kerhervé, P. The missing ocean plastic sink: gone with the rivers. *Science* **373**, 107-111, (2021).

50 Roebroek, C. T., Harrigan, S., Van Emmerik, T. H., Baugh, C., Eilander, D., Prudhomme, C. & Pappenberger, F. Plastic in global rivers: are floods making it worse? *Environmental Research Letters* **16**, 025003, (2021).

51 Jiang, X., Lu, K., Tunnell, J. W. & Liu, Z. The impacts of weathering on concentration and bioaccessibility of organic pollutants associated with plastic pellets (nurdles) in coastal environments. *Marine Pollution Bulletin* **170**, 112592, (2021).

52 Kaptan, M., Sivri, N., Blettler, M. C. & Uğurlu, Ö. Potential threat of plastic waste during the navigation of ships through the Turkish straits. *Environmental Monitoring and Assessment* **192**, 1-7, (2020).

53 Revell, L. E., Kuma, P., Le Ru, E. C., Somerville, W. R. & Gaw, S. Direct radiative effects of airborne microplastics. *Nature* **598**, 462-467, (2021).

54 Boucher, J., Friot, D. & Boucher, J. Primary microplastics in the oceans: a global evaluation of sources. *IUCN Gland, Switzerland*, pp. 46, (2017).
